# Toll-like Receptors: Key Players in Squamous Cell Carcinoma Progression

**DOI:** 10.3390/jcm13154531

**Published:** 2024-08-02

**Authors:** Jolanta Smok-Kalwat, Paulina Mertowska, Sebastian Mertowski, Stanisław Góźdź, Ewelina Grywalska

**Affiliations:** 1 Department of Clinical Oncology, Holy Cross Cancer Centre, 3 Artwińskiego Street, 25-734 Kielce, Poland; jolantasmok1@gmail.com (J.S.-K.); stanislawgozdz1@gmail.com (S.G.); 2 Department of Experimental Immunology, Medical University of Lublin, 4a Chodzki Street, 20-093 Lublin, Poland; paulina.mertowska@umlub.pl (P.M.); ewelina.grywalska@umlub.pl (E.G.); 3 Institute of Medical Science, Collegium Medicum, Jan Kochanowski University of Kielce, IX Wieków Kielc 19A, 25-317 Kielce, Poland

**Keywords:** TLR, toll-like, SCC, squamous cell carcinoma, biomarker, cancer

## Abstract

**Background/Objectives** Lung squamous cell carcinoma (SCC) is one of the major subtypes of lung cancer, characterized by diverse molecular pathways and variable clinical outcomes. This study focused on assessing the levels of TLR-2, TLR-3, TLR-4, TLR-7, TLR-8, and TLR-9 on peripheral blood lymphocytes in patients with newly diagnosed SCC compared to a group of healthy controls, in the context of disease development and patient survival, conducted over three years. The study aimed to investigate the differences in TLR expression between SCC patients and healthy people and to understand their role in the development of the disease and patient survival over three years. **Methods:** The study included the assessment of TLR-2, TLR-3, TLR-4, TLR-7, TLR-8, and TLR-9 levels on peripheral blood lymphocytes in patients with newly diagnosed SCC and in the control group. The expression of TLRs was measured using flow cytometry, and the soluble forms of the tested TLRs were measured using enzyme-linked immunosorbent assays. All the analyses were conducted over a three-year period from the time patients were recruited to the study. The obtained test results were statistically analyzed. **Results:** Results showed statistically significant differences in TLR expression between the groups, with higher TLR levels correlating with an advanced stage of disease and poorer survival rates. This suggests that the deregulation of TLR levels may be involved in promoting tumor development and influencing its microenvironment. **Conclusions:** The research, conducted over three years, indicates the need for further research on the role of TLRs in SCC, including their potential use as therapeutic targets and biomarkers. This may help to increase the effectiveness of standard treatments and improve clinical outcomes in patients with SCC.

## 1. Introduction

Despite the decline in lung cancer incidence, which has been decreasing by about 1% in women and 2.6% in men (which translates to a reduction of 32% and 56%, respectively, between 1990 and 2019), lung cancer remains one of the leading causes of death worldwide, causing 2,480,675 cases per year. In Poland, 30,379 new cases were recorded last year. Forecasts indicate that by 2045, the number of new cases worldwide will reach 4,105,589 per year. Of all lung cancer cases, about 85% are non-small cell lung cancer (NSCLC). The three main subtypes of NSCLC are squamous cell carcinoma (SCC) (25% of cases), adenocarcinoma (AC) (40% of cases), and large cell carcinoma (LCC) (10% of cases) [1,2,3]. According to epidemiological estimates, SCC accounts for 25–30% of all NSCLC cases and is more closely associated with tobacco smoking than other types of NSCLC. Tobacco smoking plays a key role in the carcinogenesis of SCC by introducing numerous carcinogens into the body that damage the DNA of airway epithelial cells. This damage leads to mutations in the genes responsible for controlling cell growth and division, which promotes malignant transformation. Tobacco smoke also causes chronic inflammation in the airways, which further increases the risk of developing SCC. As a result, smokers have a significantly higher risk of developing SCC compared to nonsmokers, which highlights the importance of preventive measures, such as smoking cessation, in preventing this form of lung cancer. Additional risk factors for the development of SCC, according to many researchers, are alcohol consumption and infection with the human papillomavirus (HPV) or Epstein–Barr virus (EBV) [4,5]. Characteristic symptoms of NSCLC, including SCC, are cough, hemoptysis, shortness of breath, wheezing, chest pain, fatigue, and recurring pneumonia and weight loss, as well as loss of appetite and fatigue [6,7]. The most commonly used diagnostic method for obtaining a definite histological diagnosis is white light bronchoscopy (WLB), as also indicated by recent autofluorescence bronchoscopy (AFI), which has 30% greater sensitivity than WLB [8]. Due to the typical central localization of lesions, the cytological examination of sputa is more significant in SCC compared to other subtypes of NSCLCs, despite the fact that its sensitivity in early lung cancer is only in the 20–30% range. In terms of radiographic diagnosis, the sensitivity of spiral CT for SCC is significantly lower than for peripherally located tumors [9].

Due to the need to distinguish SCC from other lung cancers, including small cell lung cancer (SCLC) and other NSCLCs, most patients (approximately 75%) have advanced disease (stages III/IV) at the time of diagnosis. Despite significant progress in the oncological treatment of late-stage lung cancer, survival rates remain low. The prognosis, i.e., the expected life expectancy, depends on the stage of cancer advancement, and in the case of NSCLC, the estimated 5-year survival period is Stage I—60% to 70% of patients; II degree—40% to 50% of patients; stage IIIA—less than 15% of patients; IIIB and IV degree—5% of patients [10,11]. In practice, this means that patients diagnosed with distant metastases (stage IV) have a 1-year survival rate of only 15–19% compared to 81–85% in patients diagnosed with stage I [12].

In the vast majority of patients, advanced lung cancer is diagnosed based on small biopsy and cytologic specimens. That environment necessitates the finding of predictive biomarkers to improve clinical management and patient outcomes [13,14]. Recent research has highlighted the critical role of Toll-like receptors (TLRs) within the innate immune system, underscoring their emerging significance in the intricate relationship between the tumor microenvironment and the progression of cancer [15,16,17,18]. TLRs stand out among pattern recognition receptors (PRRs) for their fundamental ability to detect and initiate responses to a variety of pathogens and danger signals [19,20]. This capability situates TLRs as pivotal elements of the first line of defense. Beyond their established function in the management of infections, TLRs have attracted interest for their potential influence on the mechanisms of tumorigenesis and the advancement of tumors [21]. At the same time, it is important to mention that many PAMPs on the surface are simultaneously detected by different TLRs. Currently, it is unclear to what extent such antigen uptake influences the level of TLR expression and therefore furthers effector functions, which is determined by the TLR correlation [22,23]. Simultaneously, evidence from preclinical investigations suggests that dysregulated TLR signaling may facilitate the growth and spread of tumor cells, as well as their ability to evade the immune system. This positions TLRs as key components within the complex web of factors that drive cancer development [24,25,26,27].

In light of these literature reports and our research team’s observations resulting from many years of experience in working with patients diagnosed with lung cancer, I would like to present the results of our research on the importance of selected TLR receptors in the pathogenesis of SCC. The main aim of our study was to determine the percentage of TLRs such as TLR-2, TLR-3, TLR-4, TLR-7, TLR-8, and TLR-9 in selected subpopulations of peripheral blood lymphocytes in patients with newly diagnosed SCC and in the group of healthy volunteers who constituted the control group. Due to the paucity of recent studies in the literature on the role of TLRs in SCC, we decided to take a closer look at the changes that occur in their expression as the disease develops and progresses, with particular emphasis on the impact on the survival of patients with SCC. The research presented in this paper illustrates our results during a 3-year follow-up of the recruited SCC patients.

## 2. Materials and Methods

### 2.1. Characteristics of Patients and Research Material

The study included 67 patients diagnosed with SCC and 40 healthy volunteers, constituting the control group. Patients were subject to several inclusion and exclusion criteria. The criteria for inclusion of patients in the study of SCC included histopathologically confirmed diagnosis of SCC, appropriate age of the patient (≥18 years), written consent of the patient to participate in the study after being fully informed about its purpose and course and availability of treatment (ability to attend follow-up visits and undergo required study procedures), no treatment prior to study entry. Exclusion criteria include the presence of another active cancer (except in some cases, e.g., basal cell carcinoma of the skin), serious comorbidities that may affect the results of the study, or patient safety (e.g., decompensated heart, liver, or kidney disease), recent receipt of other forms of anticancer treatment that may interfere with the results of the study (e.g., chemotherapy, radiotherapy), pregnancy and breastfeeding, and difficulties in understanding the purpose of the study or cooperation during its course (e.g., with mental disorders, addictions). One day before the biopsy, blood samples were collected from previously untreated patients with suspected lung cancer. Only patients in whom SCC was confirmed intraoperatively and by histopathological examination after surgery were included in the study. The control group consisted of healthy people, matched by gender and age to the study group. The health status of patients with NSCLC was confirmed by routine diagnostic tests performed during follow-up visits (at least 2 follow-up visits a year) with an internal medicine specialist and a pulmonologist. Samples for testing were collected each time during a follow-up visit. Smoking was not considered an exclusion criterion for patients participating in the study. Exclusion criteria for both groups were as follows: taking medications that affect the immune system, hormone therapy, infection within the last three months before the study, history of blood transfusion, autoimmune disease, cancer, allergies, and pregnancy or lactation within one year before this study. 

The research material consisted of 5 mL of peripheral blood collected in EDTA tubes (allowing for the assessment of the immunophenotype) and 5 mL of serum (allowing for the evaluation of the concentration of soluble forms of the tested TLRs). Each study was performed in technical duplicates. The study protocol received the approval of the Bioethics Committee of the renowned Medical University of Lublin under reference number KE-0254/283/2015.

### 2.2. Immunophenotyping

**Figure 1:**
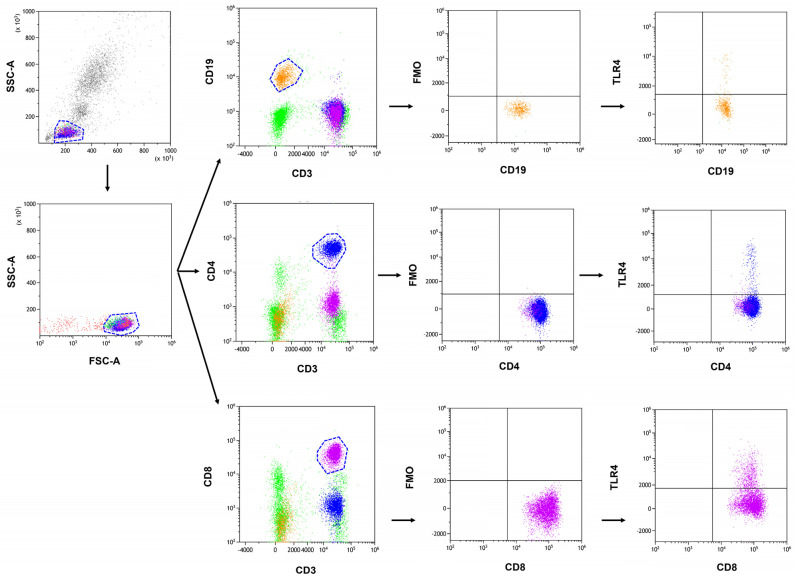
Exemplary analysis of the cells’ immunophenotype and the determination of the percentage of positive TLR expression on the example of TLR CD4+CD3+ subpopulation marked in blue, CD8+CD3+ subpopulation in violet, CD19+CD3− subpopulation in orange, and CD45+ subpopulation in green.

The analysis of lymphocyte immunophenotype in peripheral blood was performed through flow cytometry, a precise and accurate approach to cell analysis. A whole blood sample was collected and treated with a set of monoclonal human antibodies consisting of the anti-CD45 AF700, anti-CD3 PerCp, anti-CD4 BV421, anti-CD8 BV605, anti-CD19 FITC, as well as an-ti-TLR-2 APC, anti-TLR-3 PE, anti-TLR-4 PE, anti-TLR-7 PE, anti-TLR-8 APC, and an-ti-TLR-9 APC antibodies (BioLegend, San Diego, CA, USA). Subsequently, a lysing buffer was utilized to remove any red blood cells, and the remaining cells were thoroughly washed and assessed using a CytoFLEX LX instrument, a sophisticated flow cytometer (Beckman Coulter, Indianapolis, IN, USA). The resulting data were analyzed using the Kaluza Analysis program, as demonstrated in Figure 1. The CytoFLEX LX flow cytometer was subjected to daily quality control using CytoFLEX Ready to Use Daily QC Fluorospheres reagents (Beckman Coulter, Indianapolis, IN, USA).

### 2.3. Quantification of Soluble Forms of TLR Forms

To evaluate the levels of soluble TLR forms in the serum samples from all study participants, we employed enzyme-linked immunosorbent assays (ELISA). We used commercially obtained kits, specifically the Human Toll-Like Receptor 2, (TLR2) ELISA Kit (range: 109.4–7000 pg/mL; sensitivity: 17 pg/mL), Human Toll-Like Receptor 3, (TLR-3) ELISA Kit (range: 156–10,000 pg/mL; sensitivity: 10 pg/mL), and Human Toll-Like Receptor 4, (TLR-4) ELISA Kit (range: 0.41–100 ng/mL; sensitivity: 0.4 ng/well) from Abcam, Cambridge, UK. Additionally, we utilized the Human Toll-Like Receptor 7 (TLR7) ELISA Kit (range: 10–3500 ng/L; sensitivity: 5.32 ng/mL), Human Toll-Like Receptor 8 (TLR8) ELISA Kit (range: 20–0.312 ng/mL; sensitivity: 0.06 ng/mL), and Human Toll-Like Receptor 9 (TLR9) ELISA Kit (range: 20–0.312 ng/mL; sensitivity: 0.06 ng/mL) from MyBioSource, San Diego, CA, USA. We meticulously adhered to the manufacturers’ guidelines. The Victor™3 reader from PerkinElmer in Waltham, MA, USA, was used for the measurements.

### 2.4. Statistical Analysis 

The data analysis from this study was carried out using the Tibco Statistica 13.3 software, renowned for its capabilities in data analytics and visualization, based in Palo Alto, California. The Shapiro–Wilk test, a common method for assessing the normality of data distribution, was used. To compare differences among the groups, the Kruskal–Wallis test was utilized, followed by Dunn’s post hoc test for further analysis. To adjust for multiple comparisons, the Bonferroni method was applied to the *p*-values from Dunn’s test. Additionally, the study investigated the relationships between variable pairs using Spearman’s correlation coefficients. ROC curves were also employed to assess the diagnostic accuracy of laboratory tests in relation to patient parameters. To compare two groups, the Mann–Whitney U test was used. For clear and concise data presentation, GraphPad Prism software, a leading tool in scientific graphing and analysis, was used (GraphPad Prism Software v. 9.4.1, San Diego, CA, USA).

## 3. Results

### 3.1. Characteristics of SCC Patients Included in the Study at the Time of Recruitment, with Particular Emphasis on the Percentage of TLRs Tested on Selected T and B Cell Subpopulations

**Figure 2:**
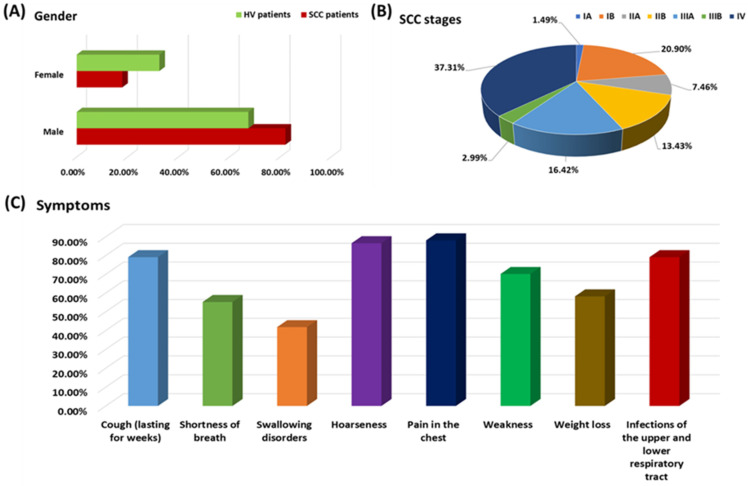
This figure shows the selected parameters characterizing the recruited patients.

The study included 67 adult patients with newly diagnosed and previously untreated SCCs confirmed by histopathological results and 40 healthy volunteers constituting the control group. The detailed criteria for the inclusion and exclusion of patients in this study are presented in detail in the Materials and Methods section and Figure 2. 

The analysis of patients according to the stage of the disease, taking gender into account, showed that stage IB predominated in women (five people), followed by IIA (four people), IV (two people), and IIB (one person). However, in the case of men, stage IV predominated (23 people), followed by stage IB (9 people), IIB (8 people), IIIA (7 people), IIA (5 people), IIIB (2 people) and IA (1 person). The most frequently reported symptoms by the SCC patients included the following: pain in the chest (88.06%); hoarseness (86.57%); cough (lasting for weeks) and infections of the upper and lower respiratory tract (79.10%); and weakness (70.15%). More than half of the recruited patients complained of weight loss exceeding 3 kg in the last 6 months (58.21%) and shortness of breath (55.22%). Additionally, 41.79% of patients complained about swallowing problems.

The analysis of selected peripheral blood morphology and biochemistry parameters and immunophenotypic assessment between the SCC patients and healthy volunteers showed several significant changes in the tested parameters, as presented in Table 1 Healthy volunteers were characterized by a statistically significant increase in lymphocytes, RBCs, and the percentage of selected immune cells in the ratio to patients with SCC. However, a significant increase in monocytes, neutrophils, and CRP was noted in patients with lung cancer.

Table 2 presents analysis of selected peripheral blood morphology and biochemistry parameters and immunophenotypic assessment between SCC patients and healthy volunteers, with particular emphasis on gender differences. No statistically significant differences in the analyzed parameters were observed between women and men with SCC. The mean values and ranges for each of the analyzed groups are detailed in the table. However, the analysis of women with SCC relative to HV women, as well as men with SCC relative to HV men, revealed a number of significant changes. Moreover, the analysis of pack–years of cigarettes smoked among women with SCC was statistically significantly higher than among women from the HV group (no such difference was observed in the case of men).

In the next step, we analyzed changes in selected peripheral blood morphology and biochemistry parameters along with immunophenotypic assessment in individual stages of SCC patients, collectively presented in Table 3. Based on the collected data, we did not observe statistically significant changes in the analyzed parameters between the individual stages of SCC patients at the time of recruitment.

We performed further analyses to determine the percentage of occurrence of individual TLR receptors, i.e., TLR-2, TLR-3, TLR-4, TLR-7, TLR-8, and TLR-9, on selected subpopulations of T lymphocytes (CD4+ and CD8+) and B lymphocytes (CD19+). The above analysis was performed between the SCC patients and healthy volunteers (Table 4), between patients with SCC and the control group while taking into account the division by gender (Table 5), and among the patients with SCC while taking into account the stage of disease development at the time of recruitment (Table 6). The results show significant differences in TLR expression between the different groups, which may suggest their role in cancer progression.

The data presented in Table 3 show that, as mentioned in the literature for patients with NSCLC, also in the case of patients with SCC, patients with lung cancer are characterized by a statistically significant increase in the percentage of lymphocytes expressing all the tested TLRs. The analysis of changes in the level of tested TLRs in the case of gender differences between the SCC patients showed statistically significant differences only in the case of CD8+TLR-9 + and sTLR-9, which was higher in men than in women (Table 5). Moreover, similarly to the previously describedcase, almost all tested TLRs and their soluble forms in serum were higher in patients with SCC than in women with HV (except for TLR2). In the case of men with SCC, they had higher TLR percentages and concentrations of soluble forms than men from the HV group (Table 5).

The data for individual stages of SCC presented in Table 6 show statistically significant differences in the parameters examined, except for CD19+TLR-2+, CD8+TLR-4+, CD19+TLR-4+, CD8+TLR-7+, and CD19+TLR-9+.

### 3.2. TLR Analysis during the 3-Year Follow-Up Period

Our research included the assessment of the analyzed parameters at the time of recruitment and after 1, 2, and 3 years of follow-up of the recruited SCC patients. A unique challenge during this period was the high mortality rate among our patients, which resulted in significant changes in the results obtained. In the first year of observation, 29 people died (43.28%; 2 women and 27 men); in the following year, another 14 (resulting in a total of 64.18% of recruited patients; 7 women and 36 men), and in the last year, another 13 (a total of 83.58% of recruited patients; 8 women and 48 men). This means that only 11 patients survived the three-year follow-up period (16.42% of recruited patients; 4 women and 7 men). The causes of death of 56 patients included infection (42.86%); multi-organ failure (26.77%); cachexia (21.43%); and coagulation disorders (8.93%).

The analysis of individual stages of patients who did not survive in each year was as follows: in the first year—16 people with IV, 2 people with IIIB (all recruited patients), 5 people with IIIA and IIIB, and 1 person with IIA; in the second year of observation—23 from IV, 2 people from IIB, 11 people with IIIA (all recruited patients), 6 people from IIB, and 1 person with IIA; and in the third year—25 people with IV (all recruited patients), 2 people with IIIB (all recruited patients), 11 people with IIIA (all recruited patients), 9 people with IIB (all recruited patients), 4 people with IIA, and 5 people with IB. This means that among the surviving patients, 1 patient was classified as IA, 9 patients for IB, and 1 for IIA.

**Figure 3:**
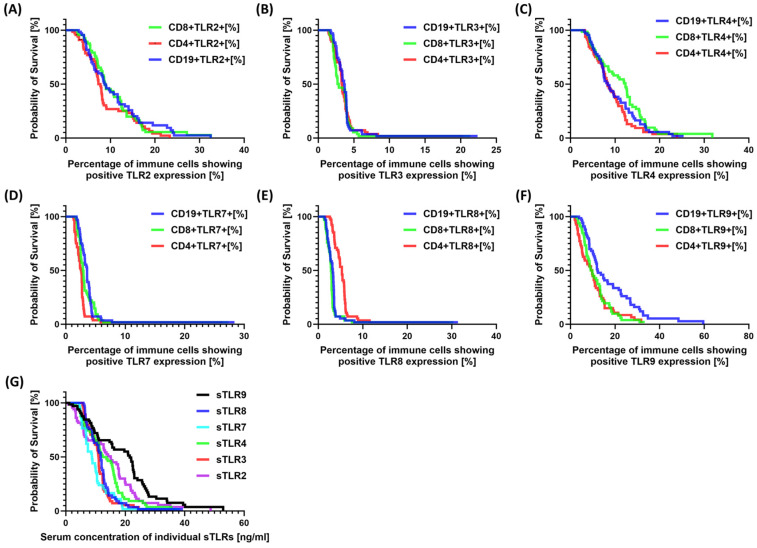
Graphical representation of Kaplan–Meier curves for individual parameters studied. (**A**) For the percentage of TLR-2 occurrence on the examined immune cell populations; (**B**) For the percentage of TLR-3 occurrence on the examined immune cell populations; (**C**) For the percentage of TLR-4 occurrence on the examined immune cell populations; (**D**) For the percentage of TLR-7 occurrence on the examined immune cell populations; (**E**) For the percentage of TLR-8 occurrence on the examined immune cell populations; (**F**) For the percentage of TLR-9 occurrence on the examined immune cell populations; (**G**) For serum sTLR concentration.

The analysis of the individual parameters of morphology, biochemistry, and immunophenotype of SCC patients in particular years is presented in Supplementary Materials Tables S1–S3. The results of the analyzed TLRs and their serum concentrations of soluble forms are presented in Supplementary Materials Tables S4 and S5. The analysis of the obtained values between patients who survived and those who died after three years of observation is presented in Table 7 and Figure 3 (Kaplan–Meier curves). No statistically significant differences were observed between deceased and living patients for CD19+TLR-2+, CD4+TLR-4+, CD8+TLR-4+, CD4+TLR-9+, CD8+TLR-9+, and CD19+TLR-9+. However, significant differences were observed for other TLRs, which may suggest their potential prognostic role in SCC.

In the next step, the results obtained at the time of recruitment were compared with the results obtained after the death of patients in selected stages of SCC, in which all patients died after a three-year follow-up period. Due to the small number of patients in stages I (15 people), II (14 people), and III (13 people), we performed a detailed analysis for patients in stage IV, which was the most numerous (25 people). The results of the collected data are presented in Table 8.

In stage IV patients, a statistically significant increase in all the analyzed TLRs in the T and B cell subpopulations and an increase in serum concentrations of all the sTLRs at the time of death compared to the time of study recruitment was demonstrated (Table 8).

### 3.3. Spearman’s Rank Correlation Analysis of Results Obtained from Analyses of the Percentage of Occurrence of Selected TLRs on Individual Lymphocyte Subpopulations and the Concentration of Their Soluble Forms in Serum

In light of the obtained research results, we decided to analyze in further stages whether the percentage of the tested TLRs correlates in individual groups of patients, taking into account their survival status and stage. For this purpose, we analyzed the results based on Sperman’s rank correlation and examined the strength of the obtained statistically significant correlation relationships. We assumed that results below 0.2 meant a weak correlation; 0.2–0.4 low correlation; 0.4–0.6 moderate correlation; 0.6–0.8 high correlation; 0.8–0.9 a very high correlation, and above 0.9 a practically full correlation.

**Figure 4:**
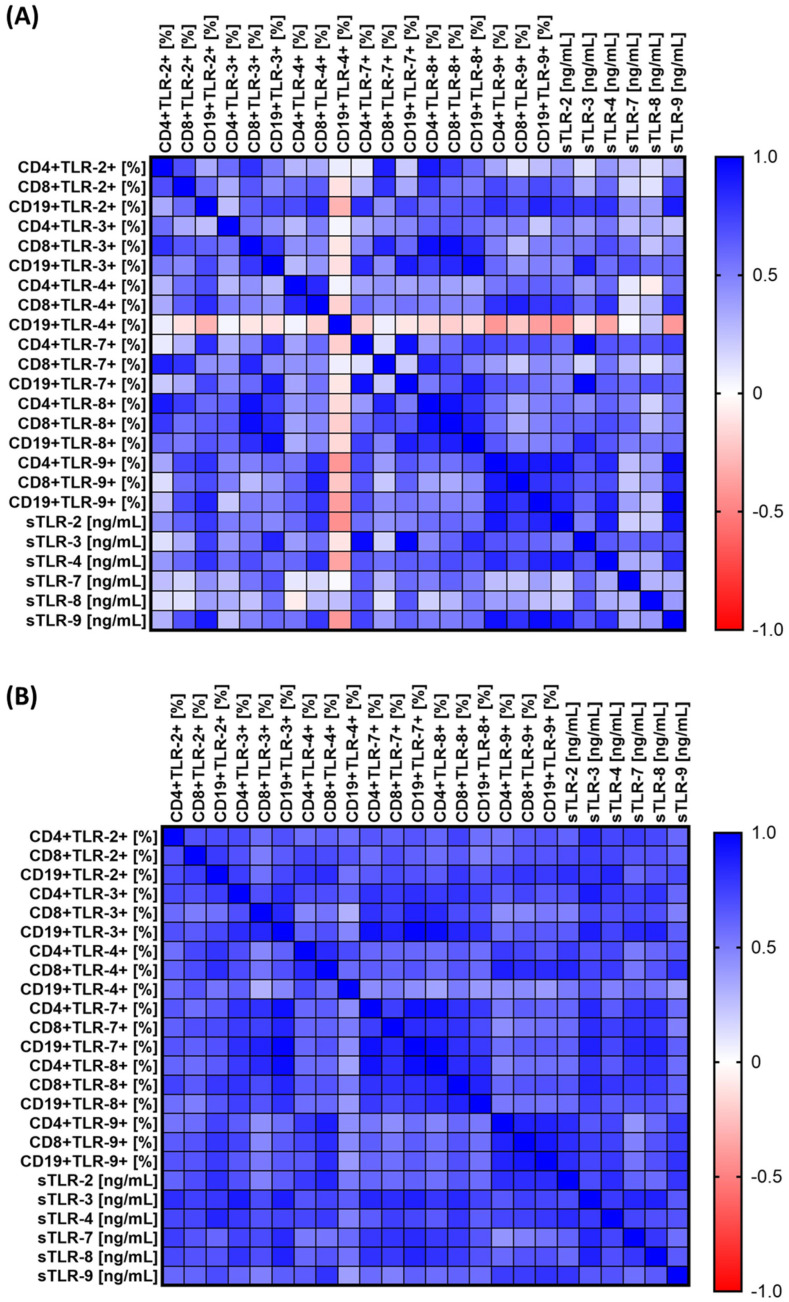
Graphical representation of Spearman’s rank correlation for SCC patients who completed the 3-year follow-up period (**A**) versus patients who died (**B**).

Among patients who survived the 3-year follow-up period, we noted 101 statistically significant positive correlations, of which 61 were high, 30 were very high correlations, and 10 were practically complete correlations (Supplementary Materials Table S7). However, in the case of patients who did not survive the observation period, we noted 276 statistically significant positive correlations, of which 5 were low, 71 were moderate, 143 were high, 50 were very high, and 7 were practically full (Supplementary Materials Table S8). A graphical representation of the observed changes is presented in the Figure 4. 

Since most of the correlations were for patients who survived the 3-year follow-up, with the most important ones included, several significant positive correlations were observed. Of note, there was a strong positive correlation (R = 0.609, *p* = 0.047) between the percentage of CD8+TLR-8+ cells and serum sTLR-3 concentration. Additionally, a significant positive correlation was found between the percentage of CD19+TLR-7+ cells and the concentration of sTLR-9 in serum (R = 0.609, *p* = 0.047), as well as between the percentage of CD8+TLR-8+ cells and the concentration of sTLR-7 in serum (R = 0.610, *p* = 0.046). Other significant correlations included a positive relationship between the percentage of CD8+TLR-2 + cells and serum sTLR-2 concentration (R = 0.618, *p* = 0.043) and between the percentage of CD4+TLR-4 + cells and CD19+TLR-9 + cells (R = 0.618, *p* = 0.043). Moreover, a significant correlation was found between the percentage of CD4+TLR-8+ and CD4+TLR-3+ cells (R = 0.618, *p* = 0.043), as well as between the percentage of CD8+TLR-9+ cells and CD19+TLR-7+ cells (R = 0.618, *p* = 0.043). Additionally, serum concentrations of various sTLRs showed significant positive correlations, e.g., sTLR-3 with sTLR-9 (R = 0.645, *p* = 0.032) and sTLR-3 with sTLR-4 (R = 0.655, *p* = 0.029). Numerous significant correlations were also observed between different TLR cell populations, e.g., CD19+TLR-2+ with CD8+TLR-3+ (R = 0.620, *p* = 0.042), CD8+TLR-2+ with CD8+TLR-4+ (R = 0.636, *p* = 0.035), and CD19+TLR7+ with CD4+TLR9+ (R = 0.664, *p* = 0.026). The most robust correlations were between CD4+TLR7+ and CD19+TLR7+ (R = 0.941, *p* = 0.000) and between CD19+TLR3+ and CD4+TLR7+ (R = 0.940, *p* = 0.000), which indicates an almost complete correlation. These results highlight the complex interaction between different TLRs and their potential impact on patient survival.

In the last analyzed group of patients in stage IV at the time of recruitment, we noted 219 positive, statistically significant correlations, of which 1 was low; 92 were moderate; 76 were high; 28 were very high; and 22 were practically full (Supplementary Materials Table S9). The analysis of the results obtained after the death of patients from this group showed the following 214 positive correlations: 92 were moderate; 81 were high; 30 were very high; and 11 were practically full (Supplementary Materials Table S10). 

Summarizing the Results section, significant differences were observed in the expression of various TLRs (TLR-2, TLR-3, TLR-4, TLR-7, TLR-8, and TLR-9) on selected T and B cell subpopulations between the patients with squamous cell carcinoma (SCC) and the healthy controls. Changes in TLR expression have also been reported between the time of recruitment and the time of death among SCC patients, suggesting their potential role in disease progression. Also, correlation analysis at various stages of the disease showed significant differences in TLR expression between the moment of recruitment and the moment of death of patients, which may indicate their role in disease progression. In stage IIB, higher TLR expression values were observed in patients at the time of death, especially for TLR-3, TLR-4, TLR-7, TLR-8, and TLR-9 on different lymphocyte subpopulations, indicating their potential prognostic importance. The obtained results are consistent with the literature data, indicating the importance of TLRs’ role in the tumor microenvironment. At the same time, the results obtained are only a starting point and more detailed studies on larger groups of patients are required to confirm the importance of TLRs in SCC and their potential application in clinical practice. Further understanding of the role of TLRs in different NSCLC subtypes may lead to the development of more personalized therapeutic approaches, improving patient outcomes and quality of life.

## 4. Discussion

In the context of cancer, it seems important to fight in the area of the tumor microenvironment, as a result of which potential molecules play a key role in the modulation of the immune response, constituting TLRs, which are a bridge between innate and adaptive immunity. At the same time, the literature reports indicate that, in the context of cancer, TLRs may influence the development of low-activity tumors characterized by low immunological activity, tumors that are more active but still more susceptible to immunotherapy. In Rolfo’s work, they indicate that TLR agonists, such as Imiquimod (TLR-7) and GSK1795091 (TLR-4), are used both as monotherapy and in combination with checkpoint inhibitors to improve patient treatment outcomes [24]. Also, in the context of cancer, it seems important to fight in the area of the tumor microenvironment, as a result of which potential molecules play a key role in the modulation of the immune response, constituting Toll-like receptors (TLRs), which are a bridge between innate and adaptive immunity. At the same time, the literature reports indicate that in the context of cancer, TLRs may influence the development of low-activity tumors characterized by low immunological activity, tumors that are more active but still more susceptible to immunotherapy. Rafolo’s work indicates that TLR agonists, such as Imiquimod (TLR-7) and GSK1795091 (TLR-4), are used both as monotherapy and in combination with checkpoint inhibitors to improve patient treatment outcomes [24]. 

At the same time, studies on a mouse model indicate that knockout of TLR 2, 4, or 9 receptors results in a lower tumor burden, and a reduction in the processes of angiogenesis and tumor cell proliferation, while increasing the process of apoptosis and changes in the tumor microenvironment [25,26]. In the above context, we searched the databases for a review of NSCLCs and found the database to be extremely large, containing approximately 90,832 articles on this topic in the PubMed and Web of Science databases at the time of writing (data from the last 24 years). At the same time, despite such a large number, only 5304 of the articles were concerned with the role of the immune system and only 59 mentioned the importance of TLRs. Since 2000, more than four times fewer articles on SCCs have been deposited (22,162), of which only 1278 were concerned with aspects of the immune response, and only a few mentioned the involvement of TLRs. This means that, despite the importance and complexity of the research regarding the participation of TLRs in SCC immunopathogenesis, the research availability is low. 

Studies on the role of TLRs in NSCLCs have provided important information on their involvement in the pathogenesis and progression of the disease, indicating that they are expressed in NSCLC tissues and cell lines. Moreover, the expression level of TLRs was higher in NSCLC compared to healthy lung tissues, which, according to researchers, provides evidence of their involvement in the disease development [27,28,29,30,31].

Some researchers have proposed that TLRs act as a double-edged sword in the tumor environment. On the one hand, TLRs expressed by immune cells may promote immune surveillance because their stimulation promotes the maturation and activation of innate and adaptive immune effectors. On the other hand, TLRs expressed by cancer cells may receive stimuli that promote tumor progression [32,33].

Although most studies in the literature focus on NSCLCs as a general group, without distinguishing between histological subtypes, some published studies indicate that the differences between them regarding TLR are not always statistically significant, which, according to many researchers, entitles them to treat the obtained results equally [34,35]. In our opinion, this is not always a good solution, because the differences between individual types of lung cancer may give us a broader picture of the mechanisms of immunopathogenesis and be used in the future to develop personalized therapies for their treatment.

Studies on the role of TLRs in NSCLCs show that activation of TLR-4 in NSCLC cells leads to increased regulation of pro-inflammatory cytokines and chemokines (TNF-α or CCL2), which contribute to the immunosuppressive nature of the tumor microenvironment [36,37,38,39,40,41]. Moreover, TLRs (especially TLR-4) have been shown to influence immune evasion mechanisms in NSCLCs. Cancer cells can use TLR signaling to suppress anti-tumor immune responses. They are leading to a reduced ability of the immune system to recognize and eliminate cancer cells [42,43]. Targeting TLR signaling pathways has emerged as a potential therapeutic strategy for NSCLC. Preclinical studies have explored the use of TLR agonists and antagonists to modulate the immune response and increase the effectiveness of treatments such as chemotherapy and immunotherapy [44,45,46]. Studies have shown that TLR expression profiles can influence the response to specific therapies in NSCLCs. For example, TLR activation has been associated with resistance to certain chemotherapeutic agents. At the same time, TLR modulation has been shown to increase the sensitivity of cancer cells to immunotherapies [47].

High TLR-4 expression has been associated with resistance to cisplatin, a commonly used chemotherapy drug in NSCLCs. TLR-4 activation in cancer cells may promote upregulation of anti-apoptotic proteins and DNA repair mechanisms, reducing sensitivity to cisplatin-induced cell death [48,49]. TLR-7 is responsible for promoting tumor progression and chemotherapy resistance, as well as, according to the study, poor clinical outcomes [50]. On the other hand, TLR-3 expression has been associated with increased sensitivity to chemotherapy in NSCLCs. TLR-3 activation in cancer cells may enhance the production of pro-apoptotic proteins and increase the susceptibility of cancer cells to chemotherapeutic agents [51,52]. Also, TLR-2 plays a tumor suppressor role in early-stage lung cancer [53]. Regarding TLR4, some studies have shown significantly higher serum levels in SCC patients [54]. When examining the role of TLRs in worsening the prognosis of SCC patients, we must emphasize that TLR signaling pathways are involved in promoting the proliferation, invasion, and metastasis of cancer cells. In particular, TLR-7 was found to promote metastasis through a mechanism involving MDSCs in a mouse model [18,30]. In the above context, we conducted analyses in which we assessed the parameters analyzed at the time of recruitment and after 1, 2, and 3 years of follow-up of patients with SCC. The analyses indicate that there was a high mortality rate, which was a significant challenge. At the same time, by analyzing the TLRs, we indicated that there were differences in TLR expression between the patients who survived and those who died. Thus, our analysis shows that higher levels of TLR-2, TLR-3, TLR-7, and TLR-8 on CD4+ T, CD8+ T, and CD19+ B cells were associated with higher mortality, which provides information that patients with increased TLR expression may have a worse prognosis. At the same time, our results also suggest that TLRs may play an ambiguous role in the tumor microenvironment. This is related to theories in which TLR expression on immune cells can support the immune response against cancer and, on the other hand, it can promote disease progression, favoring the development of inflammation or tumor immune escape.

At the same time, our analyses also fit the assumptions that TLRs may constitute targeted therapies with other treatments, such as checkpoint inhibitors, as they have shown promising results in preclinical models. This approach aims to exploit the immunomodulatory effects of TLRs to enhance the anti-tumor immune response and improve treatment outcomes [16,17,31].

In conclusion, our study assessing the role of TLRs in NSCLCs suggests a potential relationship between the stage of the disease and the TLR level, which may influence cancer progression, immune evasion, and response to treatment. At the same time, our analyses indicate a further direction for the study of TLRs as potential prognostic markers and therapeutic targets in NSCLCs, which may open new opportunities for precision medicine and tailored interventions to improve patient outcomes. At the same time, it should be mentioned that our study has some limitations due to the use of only flow cytometry and ELISA. Further research and clinical trials are needed to confirm the clinical utility of targeting TLRs in the treatment of specific NSCLC subtypes. Although our research results may help develop research on the role of TLRs in SCCs, we should consider the challenges we encountered during our research, which are undoubtedly limitations. First, the number of patients recruited for the study was small, which should be significantly expanded in future studies. Secondly, the number of deaths recorded among patients was one of the most significant obstacles recorded in this research, because most patients were late-stage patients at the time of recruitment. Therefore, their survival rate of the planned observation period was significantly shortened. Thirdly, the number of parameters examined and tested should also be increased to check a more significant part of the functioning of the immune system, which involves the need to obtain more financial resources to cover all costs, including those related to the observation process. However, we hope that the presented research will contribute, at least in some small way, to inspiring scientists to conduct further research on NSCLC subtypes and the role of TLRs in immunopathogenesis.

## 5. Conclusions

To summarize the presented data, research on the role of TLRs in SCC sheds new light on the understanding of the pathogenesis and progression of this disease. Although the literature on NSCLCs is extensive, work on the involvement of the immune system, particularly the role of TLRs, constitutes only a fraction of the available research.

Our findings point to the complex role of TLRs in the tumor environment, where they can act to benefit or disadvantage the patient depending on the context of their expression and activation. On the one hand, the expression of TLRs on immune cells may support the immune response against the tumor, on the other hand, their presence on tumor cells may promote disease progression by promoting inflammation, immune evasion, and therapy resistance.

Moreover, research has indicated the potential role of TLRs as prognostic markers and therapeutic targets, which opens opportunities for developing new treatment strategies, including targeted therapies and immunotherapies. In particular, the manipulation of TLR signaling pathways can modulate the immune response and improve the effectiveness of standard treatments such as chemotherapy.

Despite promising results, current research faces many challenges and limitations, such as a small number of study participants, limited survival rates, and the need to further expand the range of parameters studied. These limitations indicate the need for further, more detailed, and extensive clinical studies to help confirm the importance of TLRs in SCC and their potential application in clinical practice.

In the future, it will be essential to gain a deeper understanding of the differences between individual NSCLC subtypes in the context of the role of TLRs, which may contribute to the development of more personalized therapeutic approaches. Integrating discoveries with current treatment strategies has the potential to revolutionize the management of NSCLCs, offering patients better outcomes and quality of life.

## Figures and Tables

**Table 1:** Analysis of selected peripheral blood morphology and biochemistry parameters along with immunophenotypic assessment between SCC patients and healthy volunteers.

Parameters	SCC (*n* = 67)	HV (*n* = 40)	*p*-Value
	Median (Range)	Median (Range)	
WBC [10^3^/mm^3^]	6.24 (3.58–16.82)	6.22 (1.00–7.82)	0.895
LYM [10^3^/mm^3^]	1.10 (0.30–3.13)	1.85 (1.23–3.10)	0.000 *
MON [10^3^/mm^3^]	0.51 (0.28–1.18)	0.38 (0.22–0.72)	0.0001 *
NEU [10^3^/mm^3^]	4.35 (2.37–14.76)	3.45 (0.22–7.82)	0.0005 *
RBC [10^6^/mm^3^]	3.18 (2.45–3.64)	4.55 (1.78–5.19)	0.000 *
HGB [g/dL]	9.04 (6.26–11.36)	6.49 (3.95–8.40)	0.0004
PLT [10^3^/mm^3^]	219.64 (84.32–458.32)	175.11 (4.63–290.35)	0.0000 *
CRP [mg/L]	14.65 (0.50–107.63)	0.16 (0.00–1.95)	0.0000 *
T CD3+ [%]	47.44 (28.73–61.81)	71.94 (65.09–96.34)	0.0000 *
T CD3+CD8+ [%]	20.18 (6.62–39.93)	26.91 (20.18–31.07)	0.0000 *
T CD3+CD4+ [%]	27.27 (10.87–38.76)	46.76 (20.18–65.51)	0.0000 *
Ratio T CD3+CD4+ do T CD3+CD8+	1.41 (0.46–5.58)	1.78 (1.53–2.23)	0.0000 *
B CD19+ [%]	5.23 (1.31–50.62)	12.46 (7.13–16.83)	0.0000 *

* Statistically significant results.

**Table 2:** Analysis of selected peripheral blood morphology and biochemistry parameters and immunophenotypic assessment between SCC patients and healthy volunteers, with particular emphasis on gender differences.

Parameters	SCC (*n* = 67)		HV (*n* = 40)		*p*-Value			
	Male (*n* = 55) (Group 1)	Female (*n* = 12) (Group 2)	Male (*n* = 27) (Group 3)	Female (*n* = 13) (Group 4)	1 vs. 2	1 vs. 3	2 vs. 4	3 vs. 4
	Median (Range)	Median (Range)	Median (Range)	Median (Range)				
WBC [10^3^/mm^3^]	6.25 (3.58–16.82)	6.36 (4.74–7.51)	6.27 (4.95–7.78)	6.11 (4.75–7.82)	0.916	0.753	0.538	0.685
LYM [10^3^/mm^3^]	1.15 (0.52–3.13)	0.95 (0.30–1.63)	1.85 (1.23–2.85)	1.81 (1.32–3.10)	0.149	0.000 *	0.000 *	0.478
MON [10^3^/mm^3^]	0.50 (0.28–1.18)	0.56 (0.33–0.65)	0.41 (0.22–0.72)	0.37 (0.24–0.67)	1.00	0.003 *	0.045 *	0.080 *
NEU [10^3^/mm^3^]	4.22 (2.37–14.76)	4.50 (3.16–6.41)	3.48 (2.34–7.82)	3.18 (1.78–4.87)	0.577	0.039 *	0.002 *	0.024 *
RBC [10^6^/mm^3^]	3.15 (2.52–3.64)	3.24 (2.45–3.59)	4.58 (3.95–5.19)	4.54 (4.08–5.10)	0.656	0.000 *	0.000 *	0.000 *
HGB [g/dL]	9.04 (6.26–11.36)	9.01 (6.39–0.27)	6.49 (4.63–8.40)	6.69 (6.19–8.10)	0.980	0.000 *	0.000 *	0.063
PLT [10^3^/mm^3^]	220.32 (84.32–458.32)	218.62 (150.28–392.36)	177.13 (104.67–290.35)	174.61 (104.67–249.59)	0.827	0.011 *	0.018 *	0.123
CRP [mg/L]	15.01 0.50–107.63	13.47 (3.62–68.50)	0.15 (0.00–1.95)	0.23 (0.03–1.53)	0.599	0.000 *	0.000 *	0.000 *
T CD3+ [%]	47.56 (13.87–61.81)	48.75 (33.20–96.44)	70.98 (65.09–96.34)	72.41 (68.05–94.58)	0.649	0.000 *	0.000 *	0.000 *
T CD3+CD8+ [%]	17.79 (6.09–39.93)	20.98 (14.53–30.11)	26.43 (20.18–30.83)	27.52 (22.25–31.07)	0.335	0.001 *	0.000 *	0.000 *
T CD3+CD4+ [%]	27.27 (7.05–38.76)	25.08 (17.52–29.08)	46.04 (42.25–65.51)	47.69 (42.19–64.31)	0.993	0.000 *	0.000 *	0.000 *
Ratio T CD3+CD4+ do T CD3+CD8+	0.98 (0.31–3.80)	0.82 (0.47–8.37)	1.78 (1.53–2.23)	1.78 (1.53–2.13)	0.765	0.000 *	0.001 *	0.001 *
B CD19+ [%]	5.11 (1.31–50.62)	6.86 (2.46–50.62)	12.19 (7.81–16.53)	12.77 (11.03–16.83)	0.360	0.000 *	0.000 *	0.000 *

* Statistically significant results.

**Table 3:** Analysis of selected peripheral blood morphology, biochemistry parameters, and immunophenotypic assessment between stages in patients with SCC.

Parameters	I (*n* = 15)	II (*n* = 14)	III (*n* = 13)	IV (*n* = 25)	*p*-Value
	Median (Range)	Median (Range)	Median (Range)	Median (Range)	
WBC [10^3^/mm^3^]	5.64 (3.58–7.51)	6.22 (4.75–17.08)	6.47 (5.08–16.82)	6.43 (3.70–11.72)	0.5161
LYM [10^3^/mm^3^]	1.08 (0.30–1.73)	1.27 (0.55–2.65)	1.02 (0.48–3.13)	0.94 (0.52–2.22)	0.2871
MON [10^3^/mm^3^]	0.48 (0.28–1.18)	0.50 (0.29–0.88)	0.57 (0.33–1.12)	0.51 (0.33–0.95)	0.5042
NEU [10^3^/mm^3^]	3.47 (2.37–6.41)	4.59 (2.62–10.57)	4.35 93.35–14.76)	4.63 (2.41–9.01)	0.4404
RBC [10^6^/mm^3^]	3.25 (2.45–3.64)	3.31 (2.53–3.56)	3.08 (2.52–3.59)	3.03 (2.54–3.64)	0.7720
HGB [g/dl]	9.25 (6.39–10.74)	9.49 (6.87–11.36)	8.91 (6.46–10.27)	8.70 (6.26–10.95)	0.1379
PLT [10^3^/mm^3^]	219.64 (84.32–392.36)	193.12 (111.52–316.88)	239.36 (146.88–458.32)	236.64 (114.92–392.36)	0.500
CRP [mg/L]	11.27 (3.08–26.90)	11.96 (1.86–107.63)	23.17 (6.00–91.41)	11.75 (0.50–62.37)	0.3624
T CD3+ [%]	47.05 (33.20–59.34)	47.46 (30.73–60.55)	47.72 (39.29–54.350	47.44 (28.73–61.81)	0.9441
T CD3+CD8+ [%]	17.16 (6.62–39.94)	18.95 (11.10–37.99)	20.98 (8.80–30.11)	18.90 (7.44–38.92)	0.9441
T CD3+CD4+ [%]	29.82 (15.64–36.94)	26.76 (17.06–33.03)	27.27 (20.75–37.73)	27.53 (10.87–38.76)	0.8699
Ratio T CD3+CD4+ do T CD3+CD8+	1.49 (0.46–5.58)	1.38 (0.51–2.79)	1.40 (0.70–3.96)	1.42 (0.51–4.20)	0.9441
B CD19+ [%]	6.79 (2.46–13.93)	5.97 (2.01–50.62)	3.58 (1.65–42.58)	4.37 (1.31–14.56)	0.0842

**Table 4:** Analysis of the percentage of occurrence of individual TLRs on selected T and B lymphocyte subpopulations and their serum concentration of soluble forms between SCC and HV patients.

Parameters	SCC (*n* = 67)	HV (*n* = 40)	*p*-Value
	Median (Range)	Median (Range)	
T CD4+TLR-2+ [%]	3.79 (1.43–14.29)	0.83 (0.00–2.15)	0.000 *
T CD8+TLR-2+ [%]	5.52 (2.16–14.91)	0.56 (0.10–2.91)	0.000 *
B CD19+TLR-2+ [%]	4.77 (2.69–12.29)	1.32 (0.10–2.61)	0.000 *
T CD4+TLR-3+ [%]	1.88 (1.33–3.34)	1.06 (0.03–2.13)	0.000 *
T CD8+TLR-3+ [%]	1.84 (1.12–2.71)	0.67 (0.03–1.83)	0.000 *
B CD19+TLR-3+ [%]	2.17 (1.44–3.00)	0.52 (0.12–1.12)	0.000 *
T CD4+TLR-4+ [%]	4.58 (2.46–11.83)	1.08 (0.12–2.01)	0.000 *
T CD8+TLR-4+ [%]	5.48 (2.47–12.78)	0.99 (0.01–1.79)	0.000 *
B CD19+TLR-4+ [%]	4.64 (2.58–17.09)	0.79 (0.01–1.82)	0.000 *
T CD4+TLR-7+ [%]	1.49 (0.97–2.37)	0.55 (0.02–1.20)	0.000 *
T CD8+TLR-7+ [%]	1.90 (0.83–3.04)	0.32 (0.02–1.26)	0.000 *
B CD19+TLR-7+ [%]	2.18 (1.52–2.73)	0.44 (0.02–0.74)	0.000 *
T CD4+TLR-8+ [%]	3.22 (2.01–4.37)	0.76 (0.08–1.77)	0.000 *
T CD8+TLR-8+ [%]	1.63 (0.88–2.77)	0.33 (0.06–0.93)	0.000 *
B CD19+TLR-8+ [%]	1.87 (0.82–2.91)	0.56 (0.06–1.33)	0.000 *
T CD4+TLR-9+ [%]	4.92 (1.77–14.48)	0.87 (0.14–2.28)	0.000 *
T CD8+TLR-9+ [%]	5.74 (2.58–14.00)	1.41 (0.15–2.31)	0.000 *
B CD19+TLR-9+ [%]	7.58 (3.75–25.96)	1.59 (0.15–3.68)	0.000 *
sTLR-2	5.79 (0.80–19.35)	2.63 (0.16–3.96)	0.000 *
sTLR-3	5.98 (5.09–11.89)	1.47 (0.04–2.97)	0.000 *
sTLR-4	5.87 (2.74–16.02)	3.26 (0.04–3.94)	0.000 *
sTLR-7	4.90 (3.88–10.10)	1.07 (0.06–1.93)	0.000 *
sTLR-8	6.44 (4.07–12.14)	1.01 (0.01–1.98)	0.000 *
sTLR-9	9.55 (0.88–21.56)	3.21 (0.01–3.92)	0.000 *

* Statistically significant results.

**Table 5:** Analysis of the percentage of occurrence of individual TLRs on selected T and B lymphocyte subpopulations and their serum concentration of soluble forms between patients with SCC and HV, with particular emphasis on differences between genders.

Parameters	SCC (*n* = 67)		HV (*n* = 40)		*p*-Value			
	Male (*n* = 55) (Group 1)	Female (*n* = 12) (Group 2)	Male (*n* = 27) (Group 3)	Female (*n* = 13) (Group 4)				
	Median (Range)	Median (Range)	Median (Range)	Median (Range)	1 vs.2	1 vs. 3	2 vs. 4	3 vs. 4
T CD4+TLR-2+ [%]	3.82 (1.43–14.29)	3.69 (1.80–8.64)	1.04 (0.11–2.15)	0.59 (0.21–1.89)	0.432	0.000 *	0.000 *	0.000 *
T CD8+TLR-2+ [%]	5.59 (2.16–14.91)	4.89 (2.69–8.67)	0.41 (0.10–2.91)	1.06 (0.19–2.20)	0.442	0.000 *	0.000 *	0.000 *
B CD19+TLR-2+ [%]	4.98 (2.69–12.29)	4.06 (2.81–10.68)	1.43 (0.50–2.61)	1.23 (0.45–1.85)	0.144	0.000 *	0.000 *	0.000 *
T CD4+TLR-3+ [%]	1.99 (1.35–3.34)	1.75 (1.33–2.93)	1.12 (0.03–2.13)	0.96 (0.20–1.47)	0.159	0.000 *	0.000 *	0.000 *
T CD8+TLR-3+ [%]	1.86 (1.12–2.71)	1.84 (1.37–2.60)	0.54 (0.12–1.64)	0.77 (0.12–1.83)	0.840	0.000 *	0.000 *	0.000 *
B CD19+TLR-3+ [%]	2.19 (1.44–3.00)	2.02 (1.50–2.67)	0.52 (0.12–1.12)	0.52 (0.12–0.74)	0.135	0.000 *	0.000 *	0.000 *
T CD4+TLR-4+ [%]	4.66 (2.46–11.83)	4.26 (3.03–7.46)	1.20 (0.15–2.01)	1.02 (0.14–1.50)	0.481	0.000 *	0.000 *	0.000 *
T CD8+TLR-4+ [%]	5.69 (2.47–12.78)	4.85 (3.021–0.36)	1.06 (0.01–1.78)	0.80 (0.08–1.79)	0.432	0.000 *	0.000 *	0.000 *
B CD19+TLR-4+ [%]	4.41 (2.58–17.09)	5.06 (3.83–9.19)	0.79 (0.17–1.82)	0.78 (0.17–1.60)	0.266	0.000 *	0.000 *	0.000 *
T CD4+TLR-7+ [%]	1.51 (0.97–2.37)	1.39 (1.14–1.96)	0.55 (0.02–1.20)	0.55 (0.11–0.75)	0.213	0.000 *	0.000 *	0.000 *
T CD8+TLR-7+ [%]	1.92 (0.83–3.04)	1.71 (1.40–2.42)	0.32 (0.02–1.21)	0.31 (0.08–1.26)	0.395	0.000 *	0.000 *	0.000 *
B CD19+TLR-7+ [%]	2.20 (1.52–2.73)	1.97 (1.61–2.56)	0.38 (0.08–0.74)	0.54 (0.41–0.74)	0.144	0.000 *	0.000 *	0.000 *
T CD4+TLR-8+ [%]	3.32 (2.01–4.37)	3.02 (2.13–3.84)	0.77 (0.09–1.47)	0.61 (0.22–1.77)	0.185	0.000 *	0.000 *	0.000 *
T CD8+TLR-8+ [%]	1.74 (0.88–2.77)	1.50 (1.05–2.58)	0.30 (0.06–0.90)	0.41 (0.06–0.93)	0.140	0.000 *	0.000 *	0.000 *
B CD19+TLR-8+ [%]	1.98 (0.82–2.91)	1.69 (1.25–2.67)	0.59 (0.14–1.33)	0.52 (0.14–0.84)	0.144	0.000 *	0.000 *	0.000 *
T CD4+TLR-9+ [%]	4.99 (1.77–14.48)	3.21 (1.85–8.40)	0.82 (0.19–1.88)	1.03 (0.49–2.28)	0.081	0.000 *	0.000 *	0.000 *
T CD8+TLR-9+ [%]	6.37 (2.58–14.00)	4.57 (3.35–9.59)	1.26 (0.15–2.08)	1.48 (0.57–2.31)	0.040 *	0.000 *	0.000 *	0.000 *
B CD19+TLR-9+ [%]	7.67 (3.75–25.96)	5.87 (3.92–17.81)	1.58 (0.16–3.60)	1.77 (1.01–3.68)	0.164	0.000 *	0.000 *	0.000 *
sTLR-2	6.21 (0.80–19.35)	3.61 (1.86–12.05)	2.55 (0.45–3.83)	3.03 (0.92–3.96)	0.097	0.000 *	0.122	0.000 *
sTLR-3	6.09 (5.09–11.89)	5.75 (5.22–9.17)	1.43 (0.04–2.93)	1.50 (0.86–2.97)	0.135	0.000 *	0.000 *	0.000 *
sTLR-4	5.97 (2.74–16.02)	5.31 (2.86–8.46)	3.24 (0.91–3.94)	3.33 (1.76–3.89)	00.073	0.000 *	0.000 *	0.000 *
sTLR-7	4.99 (3.88–10.10)	4.76 (3.98–7.19)	1.06 (0.09–1.93)	1.08 (0.06–1.91)	0.266	0.000 *	0.000 *	0.000 *
sTLR-8	6.53 (4.07–12.14)	6.13 (4.96–7.34)	1.01 (0.01–1.98)	0.73 (0.34–1.92)	0.062	0.000 *	0.000 *	0.000 *
sTLR-9	10.01 (0.88–21.56)	6.10 (2.28–15.90)	3.20 (2.03–3.92)	3.22 (2.05–3.92)	0.032 *	0.000 *	0.000 *	0.000 *

* Statistically significant results.

**Table 6:** Analysis of the percentage of occurrence of individual TLRs on selected T and B lymphocyte subpopulations and their serum concentration of soluble forms at individual stages in SCC patients.

Parameters	I (*n* = 15)	II (*n* = 14)	III (*n* = 13)	IV (*n* = 25)	*p*-Value
	Median (Range)	Median (Range)	Median (Range)	Median (Range)	
T CD4+TLR-2+ [%]	2.47 (11.51–3.58)	3.17 (1.47–4.56)	3.85 (1.43–6.69)	5.95 (1.46–14.29)	0.000 *
T CD8+TLR-2+ [%]	3.89 (2.67–7.93)	4.75 (2.92–6.68)	5.88 (2.45–14.91)	7.77 (2.16–13.09)	0.006 *
B CD19+TLR-2+ [%]	4.06 (3.07–10.64)	4.00 (2.79–12.29)	4.47 (2.81–9.33)	9.82 (2.69–12.21)	0.123
T CD4+TLR-3+ [%]	1.58 (1.33–2.05)	1.73 (1.40–2.21)	1.79 (1.37–2.35)	2.67 (1.80–3.34)	0.002 *
T CD8+TLR-3+ [%]	1.58 (1.41–1.96)	1.77 (1.12–2.10)	2.11 (1.37–2.67)	2.06 (1.48–2.71)	0.007 *
B CD19+TLR-3+ [%]	1.67 (1.46–1.92)	1.93 (1.44–2.48)	2.17 (1.76–2.49)	2.60 (2.22–3.00)	0.000 *
T CD4+TLR-4+ [%]	4.33 (2.46–6.43)	3.77 (3.13–5.39)	4.53 (3.18–8.26)	7.46 (2.83–11.83)	0.010 *
T CD8+TLR-4+ [%]	4.98 (3.02–9.38)	4.57 (2.47–10.64)	4.76 (3.16–12.06)	8.29 (3.64–12.78)	0.128
B CD19+TLR-4+ [%]	4.60 (2.58–7.42)	4.26 (2.69–6.83)	4.60 (2.95–7.02)	7.99 (2.59–17.09)	0.606
T CD4+TLR-7+ [%]	1.22 (0.97–1.35)	1.35 (1.06–1.62)	1.49 (1.29–1.65)	1.86 (1.40–2.37)	0.000 *
T CD8+TLR-7+ [%]	1.56 (0.90–2.37)	1.91 (0.83–2.43)	1.84 (1.63–2.43)	2.02 (1.75–3.04)	0.193
B CD19+TLR-7+ [%]	11.73 (1.54–1.94)	1.93 (1.52–2.36)	2.15 (1.77–2.37)	2.49 (2.21–2.73)	0.000 *
T CD4+TLR-8+ [%]	2.34 (2.03–2.88)	2.83 (2.01–3.52)	3.22 (2.65–3.56)	3.69 (2.82–4.37)	0.000 *
T CD8+TLR-8+ [%]	1.2 (0.92–11.44)	1.45 (0.88–2.31)	1.61 (1.25–2.14)	2.33 (1.77–2.77)	0.000 *
B CD19+TLR-8+ [%]	1.39 (0.99–2.18)	1.63 (0.92–2.31)	1.87 (1.48–2.33)	2.47 (0.82–2.91)	0.000 *
T CD4+TLR-9+ [%]	4.17 (1.81–12.71)	3.54 (1.77–14.26)	2.87 (1.85–9.54)	6.63 (2.24–14.48)	0.009 *
T CD8+TLR-9+ [%]	4.95 (3.44–8.32)	4.64 (2.58–9.72)	4.57 (3.29–12.44)	8.29 (4.57–4.00)	0.029 *
B CD19+TLR-9+ [%]	6.59 (4.42–14.75)	6.73 (4.67–21.50)	6.05 (3.75–22.00)	10.22 (4.74–25.96)	0.057
sTLR-2	5.10 (1.93–12.05)	3.70 (0.80–15.61)	4.47 (3.07–17.13)	11.27 (1.51–19.35)	0.014 *
sTLR-3	5.39 (5.09–5.60)	5.64 (5.09–6.47)	5.95 (5.49–6.64)	7.82 (6.15–11.89)	0.000 *
sTLR-4	5.25 (2.86–9.38)	5.09 (2.74–12.35)	5.61 (4.23–14.59)	11.38 (4.53–16.02)	0.002 *
sTLR-7	4.27 (3.91–7.19)	4.72 (3.88–7.18)	4.86 (4.36–5.09)	5.19 (5.00–10.10)	0.000 *
sTLR-8	5.79 (4.07–9.00)	6.33 (5.72–7.91)	6.39 (5.99–6.98)	7.36 (5.94–12.14)	0.000 *
sTLR-9	8.55 (2.99–17.50)	8.90 (2.26–16.26)	4.69 (2.28–18.92)	14.83 (0.88–21.56)	0.016 *

* Statistically significant results.

**Table 7:** Analysis of the percentage of occurrence of individual TLRs in selected T and B lymphocyte subpopulations and the concentration of their soluble forms in serum between living and dead SCC patients.

Parameters	Dead SCC Patients after 3 Years	Alive SCC Patients after 3 Years	*p*-Value
	Median (Range)	Median (Range)	
T CD4+TLR-2+ [%]	7.31 (1.49–23.31)	3.40 (2.41–17.37)	0.0001 *
T CD8+TLR-2+ [%]	8.65 (2.55–32.48)	4.93 (3.49–18.37)	0.005 *
B CD19+TLR-2+ [%]	8.40 (2.91–32.48)	5.18 (3.73–19.37)	0.264
T CD4+TLR-3+ [%]	3.28 (1.43–8.36)	1.99 (1.78–20.37)	0.003 *
T CD8+TLR-3+ [%]	2.71 (1.56–5.67)	1.95 (1.83–21.37)	0.002 *
B CD19+TLR-3+ [%]	3.63 (1.75–8.19)	2.05 (1.93–22.37)	0.000 *
T CD4+TLR-4+ [%]	8.12 (3.28–23.92)	6.03 (3.11–23.37)	0.052
T CD8+TLR-4+ [%]	11.47 (2.57–31.84)	6.29 (3.20–24.37)	0.118
B CD19+TLR-4+ [%]	7.87 (3.07–22.90)	5.11 (3.26–25.37)	0.008 *
T CD4+TLR-7+ [%]	2.48 (1.29–5.86)	1.54 (1.02–26.37)	0.001 *
T CD8+TLR-7+ [%]	2.87 (1.65–6.33)	2.02 (1.88–27.37)	0.028 *
B CD19+TLR-7+ [%]	3.56 (1.82–7.79)	2.17 (2.03–28.37)	0.0004 *
T CD4+TLR-8+ [%]	5.30 (2.58–11.53)	3.00 (2.71–29.37)	0.001 *
T CD8+TLR-8+ [%]	2.89 (1.29–7.60)	1.50 (1.37–30.37)	0.000 *
B CD19+TLR-8+ [%]	3.23 (1.46–8.11)	1.73 (1.61–31.37)	0.000 *
T CD4+TLR-9+ [%]	8.02 (1.94–31.70)	5.27 (3.04–32.37)	0.646
T CD8+TLR-9+ [%]	8.64 (2.68–30.99)	7.25 (3.65–33.37)	0.310
B CD19+TLR-9+ [%]	11.88 (3.90–59.54)	8.33 (4.71–34.37)	0.229
sTLR-2	13.43 (0.83–48.75)	6.48 (2.44–35.37)	0.095
sTLR-3	10.94 (5.64–24.52)	6.77 (5.87–36.37)	0.005 *
sTLR-4	11.81 (2.85–35.51)	6.43 (3.62–37.37)	0.024 *
sTLR-7	8.65 (4.48–19.10)	5.37 (4.76–38.37)	0.020 *
sTLR-8	11.67 (5.95–24.28)	6.84 (6.13–39.37)	0.002 *
sTLR-9	20.31 (1.18–52.94)	13.35 (3.17–40.37)	0.170

* Statistically significant results.

**Table 8:** Analysis of the percentage of occurrence of individual TLRs in selected T and B lymphocyte subpopulations and the concentration of their soluble forms in serum between living and dead patients with stage IV SCC.

Parameters	SCC Patients in IV Stage in Recrutation Time	Patients with Stage IV SCC Died after 3 Years of Follow-Up	*p*-Value
	Median (Range)	Median (Range)	
T CD4+TLR-2+ [%]	5.95 (1.46–14.29)	7.98 (1.96–21.30)	0.0143 *
T CD8+TLR-2+ [%]	7.77 (2.16–13.09)	8.83 (2.89–32.48)	0.0389 *
B CD19+TLR-2+ [%]	9.82 (2.69–12.21)	11.64 (3.61–32.48)	0.0189 *
T CD4+TLR-3+ [%]	2.67 (1.80–3.34)	3.36 (2.41–8.36)	0.000 *
T CD8+TLR-3+ [%]	2.06 (1.48–2.71)	2.71 (2.08–5.67)	0.000 *
B CD19+TLR-3+ [%]	2.60 (2.22–3.00)	3.43 (2.67–8.19)	0.000 *
T CD4+TLR-4+ [%]	7.46 (2.83–11.83)	10.74 (3.79–23.92)	0.0045 *
T CD8+TLR-4+ [%]	8.29 (3.64–12.78)	12.67 (4.88–31.84)	0.0008 *
B CD19+TLR-4+ [%]	7.99 (2.59–17.09)	12.34 (4.23–22.90)	0.0107 *
T CD4+TLR-7+ [%]	1.86 (1.40–2.37)	2.41 (1.46–5.86)	0.000 *
T CD8+TLR-7+ [%]	2.02 (1.75–3.04)	2.86 (2.05–6.33)	0.000 *
B CD19+TLR-7+ [%]	2.49 (2.21–2.73)	3.25 (2.55–7.79)	0.000 *
T CD4+TLR-8+ [%]	3.69 (2.82–4.37)	4.91 (2.93–11.53)	0.000 *
T CD8+TLR-8+ [%]	2.33 (1.77–2.77)	2.99 (2.19–7.60)	0.000 *
B CD19+TLR-8+ [%]	2.47 (0.82–2.91)	3.26 (1.84–8.11)	0.000 *
T CD4+TLR-9+ [%]	6.63 (2.24–14.48)	9.72 (3.01–31.70)	0.0079 *
T CD8+TLR-9+ [%]	8.29 (4.57–14.00)	9.78 (5.87–30.99)	0.016 *
B CD19+TLR-9+ [%]	10.22 (4.74–25.96)	13.68 (6.35–59.54)	0.0084 *
sTLR-2	11.27 (1.51–19.35)	17.59 (2.02–48.75)	0.0070 *
sTLR-3	7.82 (6.15–11.89)	10.98 (7.69–24.52)	0.000 *
sTLR-4	11.38 (4.53–16.02)	15.91 (6.07–35.51)	0.002 *
sTLR-7	5.19 (5.00–10.10)	7.43 (5.35–18.54)	0.000 *
sTLR-8	7.36 (5.94–12.14)	11.67 (7.04–24.28)	0.00 *
sTLR-9	14.83 (0.88–21.56)	21.97 (1.18–52.94)	0.018 *

* Statistically significant results.

## Data Availability

All necessary information regarding the preparation of this work is available on written request from the corresponding author.

## Supplementary Materials

The following supporting information can be downloaded at: https://www.mdpi.com/article/10.3390/jcm13154531/s1, Table S1. Analysis of selected peripheral blood morphology and biochemistry parameters along with immunophenotypic assessment after 1-year of observation; Table S2. Analysis of selected peripheral blood morphology and biochemistry parameters along with immunophenotypic assessment after 2 years of observations; Table S3. Analysis of selected peripheral blood morphology and biochemistry parameters along with immunophenotypic assessment after 3 years of observations; Table S4. Analysis of the percentage of occurrence of individual TLRs on selected T and B lymphocyte subpopulations and their serum concentration of soluble forms after 1 year of observation; Table S5. Analysis of the percentage of occurrence of individual TLRs on selected T and B lymphocyte subpopulations and their serum concentration of soluble forms after 2 years of observations; Table S6. Analysis of the percentage of occurrence of individual TLRs in selected T and B lymphocyte subpopulations and the concentration of their soluble forms in serum between living and dead patients with stage IIIB SCC; Table S7. Spearman’s rank correlation analysis for patients who survived the 3-year follow-up period; Table S8. Spearman’s rank correlation analysis for patients who did not survive the 3-year follow-up period; Table S9. Spearman’s rank correlation analysis for stage IV patients at the time of recruitment and study; Table S10. Spearman’s rank correlation analysis for stage IV patients at the time of death.
