# Supplementary material for: Toll-like Receptors: Key Players in Squamous Cell Carcinoma Progression
Source: J Clin Med. 2024 Aug 2;13(15):4531. doi: 10.3390/jcm13154531 (PMC11313009; doi:10.3390/jcm13154531)
Supplement: Supplementary file 1 [file jcm-13-04531-s001.zip › jcm-3124584-supplementary.pdf]

Table S1. Analysis of selected peripheral blood morphology and biochemistry parameters along with immunophenotypic assessment after 1-year of observation

Parameters	Dead SCC patients after 1 year<br>Median (Range)	Alive SCC patients after 1 year<br>Median (Range)	p-vaule
WBC [$10^3/mm^3$]	7.86 (3.85-60.00)	6.56 (3.72-16.87)	0.088
LYM [$10^3/mm^3$]	1.20 (0.57-17.49)	1.33 (0.37-3.50)	0.151
MON [$10^3/mm^3$]	0.65 (0.30-2.98)	0.55 (0.29-1.47)	0.1377
NEU [$10^3/mm^3$]	5.53 (2.76-15.35)	4.31 (2.46-12.97)	0.027*
RBC [$10^6/mm^3$]	3.65 (2.62-15.35)	3.45 (2.69-4.58)	0.304
HGB [g/dl]	10.84 (6.72-12.85)	10.15 (7.14-13.32)	0.243
PLT [$10^3/mm^3$]	250.58 (140.03-525.76)	247.98 (87.69-524.85)	0.431
CRP [mg/L]	19.41 (1.72-95.07)	13.96 (0.71-111.94)	0.166
T CD3+ [%]	55.53 (14.43-65.76)	53.42 (17.24-74.59)	0.298
T CD3+CD8+ [%]	23.62 (7.20-40.79)	21.55 (6.82-49.51)	0.550
T CD3+CD4+ [%]	31.78 (7.33-51.94)	29.71 (8.82-36.42)	0.584
Ratio T CD3+CD4+ do T CD3+CD8+	1.11 (0.46-5.94)	1.09 (0.36-10.38)	0.517
B CD19+ [%]	6.03 (1.72-52.64)	6.72 (1.88-67.46)	0.304

Table S2. Analysis of selected peripheral blood morphology and biochemistry parameters along with immunophenotypic assessment after 2 years of observations

Parameters	Dead SCC patients after 2 year<br>Median (Range)	Alive SCC patients after 2 year<br>Median (Range)	p-vaule
WBC [$10^3/mm^3$]	6.98 (2.89-17.49)	5.26 (3.79-9.79)	0.033*
LYM [$10^3/mm^3$]	1.07 (0.34-2.98)	1.44 (0.76-3.46)	0.031*
MON [$10^3/mm^3$]	0.57 (0.22-1.19)	0.38 (0.26-1.13)	0.001*
NEU [$10^3/mm^3$]	4.93 (1.58-15.35)	3.29 (2.10-7.79)	0.002*
RBC [$10^6/mm^3$]	3.32 (1.73-4.76)	2.74 (2.09-3.41)	0.003*
HGB [g/dl]	9.62 (4.50-12.85)	7.94 (6.14-10.24)	0.055
PLT [$10^3/mm^3$]	224.67 (103.64-525.76)	180.74 (89.14-333.11)	0.01*
CRP [mg/L]	20.84 (1.72-146.39)	10.70 (0.55-85.75)	0.0014*
T CD3+ [%]	49.33 (10.86-80.79)	47.13 (30.29-65.82)	0.478
T CD3+CD8+ [%]	18.68 (4.29-51.54)	19.27 (9.05-44.29)	0.780

T CD3+CD4+ [%]	27.72 (5.56-51.94)	25.48 (15.87-40.79)	0.922
Ratio T CD3+CD4+ do T CD3+CD8+	0.84 (0.28-3.83)	0.98 (0.32-9.29)	0.352
B CD19+ [%]	3.73 (1.18-52.64)	6.53 (1.60-60.35)	0.043*

Table S3. Analysis of selected peripheral blood morphology and biochemistry parameters along with immunophenotypic assessment after 3 years of observations

Parameters	Dead SCC patients after 3 years	Alive SCC patients after 3 years	p-vaule
	Median (Range)	Median (Range)	
WBC [10^3/mm^3]	6.58 (2.04-17.49)	5.33 (4.11-7.51)	0.457
LYM [10^3/mm^3]	0.92 (0.34-2.98)	1.19 (0.30-1.73)	0.223
MON [10^3/mm^3]	0.51 (0.16-1.19)	0.40 (0.33-0.56)	0.287
NEU [10^3/mm^3]	4.68 (1.35-15.35)	3.44 (2.29-6.41)	0.243
RBC [10^6/mm^3]	3.20 (1.62-4.76)	3.04 (2.17-3.57)	0.953
HGB [g/dl]	9.12 (4.50-12.85)	9.10 (6.39-10.73)	0.980
PLT [10^3/mm^3]	196.31 (48.06-525.76)	208.57 (146.74-349.20)	0.552
CRP [mg/L]	25.30 (1.55-242.67)	14.24 (1.51-39.99)	0.044*
T CD3+ [%]	48.93 (10.86-80.79)	46.89 (33.17-59.28)	0.940
T CD3+CD8+ [%]	18.66 (4.29-51.54)	17.14 (5.54-39.89)	0.770
T CD3+CD4+ [%]	24.65 (5.56-51.94)	29.29 (17.50-35.42)	0.553
Ratio T CD3+CD4+ do T CD3+CD8+	0.80 (0.20-5.69)	0.95 (0.31-3.18)	0.361
B CD19+ [%]	3.74 (1.14-52.64)	6.42 (3.14-13.91)	0.07

Table S4. Analysis of the percentage of occurrence of individual TLRs on selected T and B lymphocyte subpopulations and their serum concentration of soluble forms after 1 year observation.

Parameters	Dead SCC patients after 1 year	Alive SCC patients after 1 year	p-vaule
	Median (Range)	Median (Range)	
T CD4+TLR-2+ [%]	6.78 (1.49-45.07)	3.54 (1.53-11.71)	0.043*
T CD8+TLR-2+ [%]	6.80 (2.55-18.73)	5.73 (3.03-14.98)	0.174
B CD19+TLR-2+ [%]	5.42 (2.91-16.78)	5.32 (3.15-15.25)	0.612
T CD4+TLR-3+ [%]	2.43 (1.43-16.36)	1.96 (1.64-4.11)	0.023*
T CD8+TLR-3+ [%]	2.29 (1.56-4.47)	1.97 (1.16-3.02)	0.030*
B CD19+TLR-3+ [%]	2.81 (1.75-4.02)	2.07 (1.50-3.79)	0.000*
T CD4+TLR-4+ [%]	5.99	5.04	0.214

	(3.28-16.36)	(3.05-11.03)	
T CD8+TLR-4+ [%]	5.95<br>(2.57-16.91)	6.15<br>(3.14-14.69)	0.710
B CD19+TLR-4+ [%]	6.83<br>(3.07-22.90)	5.08<br>(2.80-9.55)	0.013*
T CD4+TLR-7+ [%]	1.90<br>(1.29-22.90)	1.53<br>(1.00-2.71)	0.003*
T CD8+TLR-7+ [%]	2.50<br>(1.65-3.99)	2.08<br>(0.86-2.99)	0.02*
B CD19+TLR-7+ [%]	2.76<br>(1.82-3.99)	2.15<br>(1.58-3.68)	0.0002*
T CD4+TLR-8+ [%]	3.82<br>(2.58-5.86)	3.01<br>(2.09-5.44)	0.001*
T CD8+TLR-8+ [%]	2.38<br>(1.29-5.86)	1.53<br>(0.92-3.64)	0.000*
B CD19+TLR-8+ [%]	2.57<br>(1.46-3.90)	1.72<br>(0.85-3.79)	0.0001
T CD4+TLR-9+ [%]	5.60<br>(1.94-19.41)	5.44<br>(1.84-19.45)	0.646
T CD8+TLR-9+ [%]	7.21<br>(2.68-19.41)	6.26<br>(3.18-14.30)	0.368
B CD19+TLR-9+ [%]	8.49<br>(3.90-34.78)	7.94<br>(4.39-24.85)	0.322
sTLR2	6.02<br>(0.83-34.78)	6.47<br>(1.93-19.07)	0.739
sTLR3	8.25<br>(5.64-25.93)	6.63<br>(5.29-13.13)	0.027*
sTLR4	6.72<br>(2.85-17.46)	6.58<br>(3.55-16.40)	0.692
sTLR7	6.70<br>(4.48-17.46)	5.28<br>(4.04-14.55)	0.079
sTLR8	7.69<br>(5.95-14.01)	7.01<br>(4.63-12.90)	0.011*
sTLR9	9.68<br>(1.18-27.73)	7.53<br>(2.55-18.20)	0.000*

Table S5. Analysis of the percentage of occurrence of individual TLRs on selected T and B lymphocyte subpopulations and their serum concentration of soluble forms after 2 years of observations.

Parameters	Dead SCC patients after 2 years	Alive SCC patients after 2 years	p-vaule
	Median (Range)	Median (Range)	
T CD4+TLR-2+ [%]	7.23<br>(1.49-19.09)	3.69<br>(1.17-7.53)	0.000*
T CD8+TLR-2+ [%]	8.61<br>(2.55-22.31)	4.95<br>(2.54-11.48)	0.001*
B CD19+TLR-2+ [%]	6.42<br>(2.91-23.80)	6.10<br>(2.59-18.39)	0.352
T CD4+TLR-3+ [%]	3.21<br>(1.43-6.70)	2.22<br>(1.41-3.07)	0.000*
T CD8+TLR-3+ [%]	2.52<br>(1.56-4.92)	2.22<br>(0.89-2.57)	0.002*
B CD19+TLR-3+ [%]	3.39<br>(1.75-6.18)	2.33<br>(1.15-2.90)	0.000*
T CD4+TLR-4+ [%]	7.40<br>(3.28-17.10)	5.56<br>(2.49-8.80)	0.028*
T CD8+TLR-4+ [%]	8.47<br>(2.57-19.66)	6.94<br>(3.32-15.91)	0.142
B CD19+TLR-4+ [%]	7.49<br>(3.07-22.90)	5.48<br>(2.15-9.31)	0.002*
T CD4+TLR-7+ [%]	2.37	1.68	0.000*

	(1.29-4.42)	(0.85-2.07)	
T CD8+TLR-7+ [%]	2.61<br>(1.65-4.87)	2.29<br>(0.66-3.00)	0.003*
B CD19+TLR-7+ [%]	3.21<br>(1.82-6.00)	2.42<br>(1.21-2.75)	0.000*
T CD4+TLR-8+ [%]	4.87<br>(2.58-8.87)	3.36<br>(1.60-4.07)	0.000*
T CD8+TLR-8+ [%]	2.79<br>(1.29-5.94)	1.73<br>(0.70-2.69)	0.000*
B CD19+TLR-8+ [%]	3.20<br>(1.46-6.18)	1.98<br>(0.65-2.87)	0.000*
T CD4+TLR-9+ [%]	6.26<br>(1.94-31.70)	6.18<br>(1.41-21.33)	0.654
T CD8+TLR-9+ [%]	7.35<br>(2.68-22.90)	7.30<br>(2.50-14.54)	0.313
B CD19+TLR-9+ [%]	10.16<br>(3.90-40.51)	9.97<br>(4.00-27.15)	0.635
sTLR2	12.81<br>(0.83-31.08)	7.37<br>(2.11-17.23)	0.09
sTLR3	9.86<br>(5.64-21.40)	7.71<br>(4.05-8.66)	0.001*
sTLR4	10.77<br>(2.85-26.73)	7.62<br>(3.98-18.48)	0.032*
sTLR7	6.89<br>(4.48-23.72)	5.90<br>(3.09-9.02)	0.003*
sTLR8	10.06<br>(5.95-21.02)	7.45<br>(4.56-11.29)	0.0005*
sTLR9	15.65<br>(1.18-40.11)	10.14<br>(2.17-21.95)	0.014*

Table S6. Analysis of the percentage of occurrence of individual TLRs in selected T and B lymphocyte subpopulations and the concentration of their soluble forms in serum between living and dead patients with stage IIIB SCC.

**Parameters**	**SCC patients in IIIB stage in recrutation time**	**Patients with stage IIIB SCC died after 3 years of follow-up**	**p-vaule**
	**Median (Range)**	**Median (Range)**	
T CD4+TLR-2+ [%]	3.21<br>(2.83-3.60)	4.14<br>(2.20-8.33)	0.666
T CD8+TLR-2+ [%]	3.94<br>(2.60-5.28)	5.13<br>(4.01-10.63)	0.666
B CD19+TLR-2+ [%]	4.23<br>(3.24-5.22)	4.29<br>(2.91-8.37)	0.666
T CD4+TLR-3+ [%]	1.84<br>(1.68-1.99)	2.00<br>(1.46-3.07)	0.666
T CD8+TLR-3+ [%]	1.88<br>(1.75-2.00)	1.84<br>(1.56-3.76)	0.666
B CD19+TLR-3+ [%]	1.87<br>(1.76-1.99)	2.48<br>(2.16-3.97)	0.666
T CD4+TLR-4+ [%]	4.63<br>(3.95-5.31)	3.80<br>(3.28-8.16)	0.666
T CD8+TLR-4+ [%]	4.83<br>(3.93-5.73)	4.21<br>(2.57-6.86)	0.666
B CD19+TLR-4+ [%]	5.53<br>(4.03-7.02)	4.99<br>(3.30-7.65)	0.666
T CD4+TLR-7+ [%]	1.33<br>(1.29-1.38)	1.64<br>(1.46-2.73)	0.666
T CD8+TLR-7+ [%]	2.22<br>(2.06-2.39)	1.87<br>(1.83-3.12)	0.666
B CD19+TLR-7+ [%]	1.86	2.36	0.666

	(1.77-1.95)	(2.11-4.00)	
T CD4+TLR-8+ [%]	2.77<br>(2.65-2.89)	3.60<br>(3.23-5.95)	0.666
T CD8+TLR-8+ [%]	1.37<br>(1.25-1.48)	1.87<br>(1.63-3.00)	0.666
B CD19+TLR-8+ [%]	1.82<br>(1.48-2.16)	2.12<br>(1.78-3.51)	0.666
T CD4+TLR-9+ [%]	3.25<br>(2.14-4.36)	3.22<br>(1.98-4.02)	0.666
T CD8+TLR-9+ [%]	5.22<br>(3.37-7.07)	5.03<br>(2.68-8.90)	0.666
B CD19+TLR-9+ [%]	5.96<br>(3.75-8.17)	6.58<br>(5.36-8.53)	0.666
sTLR2	4.58<br>(3.07-6.10)	3.57<br>(0.83-7.36)	0.666
sTLR3	5.56<br>(5.49-5.64)	6.61<br>(6.09-11.10)	0.333
sTLR4	5.18<br>(4.75-5.61)	4.49<br>(2.85-9.66)	0.666
sTLR7	4.51<br>(4.36-4.65)	5.35<br>(4.97-18.95)	0.666
sTLR8	6.12<br>(5.99-6.26)	6.92<br>(6.44-12.58)	0.666
sTLR9	7.42<br>(4.69-10.15)	7.49<br>(2.35-27.40)	0.666

Table S7. Spearman's rank correlation analysis for patients who survived the 3-year follow-up period

Para zmiennych	R	t(N-2)	p
CD8+TLR-8+ [%] & sTLR-3 in serum [ng/mL]	0.609	2.302	0.047 *
CD19+TLR-7+ [%] & sTLR-9 in serum [ng/mL]	0.609	2.304	0.047 *
CD8+TLR-8+ [%] & sTLR-7 in serum [ng/mL]	0.610	2.310	0.046 *
CD8+TLR-2+ [%] & sTLR-2 in serum [ng/mL]	0.618	2.359	0.043 *
CD4+TLR-4+ [%] & CD19+TLR-9+ [%]	0.618	2.359	0.043 *
CD4+TLR-8+ [%] & CD4+TLR-3+ [%]	0.618	2.359	0.043 *
CD8+TLR-9+ [%] & CD19+TLR-7+ [%]	0.618	2.359	0.043 *
sTLR-2 in serum [ng/mL] & CD8+TLR-2+ [%]	0.618	2.359	0.043 *
CD19+TLR-2+ [%] & CD8+TLR-3+ [%]	0.620	2.368	0.042 *
CD8+TLR-2+ [%] & CD8+TLR-4+ [%]	0.636	2.475	0.035 *
CD19+TLR-7+ [%] & sTLR-4 in serum [ng/mL]	0.636	2.475	0.035 *
CD19+TLR-2+ [%] & CD8+TLR-8+ [%]	0.641	2.504	0.034 *

CD4+TLR-7+ [%] & sTLR-7 in serum [ng/mL]	0.643	2.519	0.033 *
CD8+TLR-9+ [%] & sTLR-3 in serum [ng/mL]	0.645	2.535	0.032 *
sTLR-3 in serum [ng/mL] & sTLR-9 in serum [ng/mL]	0.645	2.535	0.032 *
CD4+TLR-3+ [%] & CD8+TLR-8+ [%]	0.650	2.565	0.030 *
CD4+TLR-7+ [%] & sTLR-8 in serum [ng/mL]	0.653	2.586	0.029 *
sTLR-3 in serum [ng/mL] & sTLR-4 in serum [ng/mL]	0.655	2.597	0.029 *
sTLR-4 in serum [ng/mL] & sTLR-3 in serum [ng/mL]	0.655	2.597	0.029 *
CD19+TLR-8+ [%] & CD4+TLR-9+ [%]	0.658	2.618	0.028 *
CD8+TLR-2+ [%] & CD8+TLR-3+ [%]	0.661	2.640	0.027 *
CD19+TLR-8+ [%] & sTLR-4 in serum [ng/mL]	0.662	2.651	0.026 *
CD19+TLR-7+ [%] & CD8+TLR-8+ [%]	0.664	2.661	0.026 *
CD19+TLR-7+ [%] & CD4+TLR-9+ [%]	0.664	2.661	0.026 *

CD4+TLR-9+ [%] & CD19+TLR-7+ [%]	0.664	2.661	0.026 *
CD19+TLR-2+ [%] & CD19+TLR-8+ [%]	0.671	2.717	0.024 *
CD4+TLR-7+ [%] & sTLR-4 in serum [ng/mL]	0.671	2.717	0.024 *
CD8+TLR-9+ [%] & CD4+TLR-7+ [%]	0.671	2.717	0.024 *
CD4+TLR-9+ [%] & sTLR-3 in serum [ng/mL]	0.673	2.728	0.023 *
CD19+TLR-3+ [%] & sTLR-7 in serum [ng/mL]	0.676	2.751	0.022 *
CD8+TLR-2+ [%] & sTLR-9 in serum [ng/mL]	0.682	2.796	0.021 *
CD4+TLR-7+ [%] & CD19+TLR-9+ [%]	0.685	2.820	0.020 *
CD4+TLR-2+ [%] & CD8+TLR-2+ [%]	0.691	2.867	0.019 *
CD8+TLR-3+ [%] & sTLR-4 in serum [ng/mL]	0.697	2.916	0.017 *
CD19+TLR-2+ [%] & CD4+TLR-4+ [%]	0.700	2.941	0.016 *
CD8+TLR-9+ [%] & sTLR-4 in serum [ng/mL]	0.700	2.941	0.016 *
CD8+TLR-8+ [%] & sTLR-4 in serum [ng/mL]	0.705	2.981	0.015 *
CD4+TLR-7+ [%] & CD4+TLR-9+ [%]	0.708	3.006	0.015 *
CD8+TLR-2+ [%] & CD19+TLR-9+ [%]	0.709	3.017	0.015 *
CD8+TLR-9+ [%] & CD19+TLR-2+ [%]	0.709	3.017	0.015 *
CD8+TLR-2+ [%] & CD4+TLR-9+ [%]	0.718	3.096	0.013 *
CD19+TLR-2+ [%] & CD19+TLR-3+ [%]	0.720	3.111	0.013 *
CD8+TLR-7+ [%] & CD8+TLR-8+ [%]	0.723	3.141	0.012 *
CD19+TLR-2+ [%] & CD19+TLR-7+ [%]	0.727	3.179	0.011 *
CD4+TLR-7+ [%] & sTLR-9 in serum [ng/mL]	0.740	3.298	0.009 *
CD19+TLR-3+ [%] & CD4+TLR-8+ [%]	0.752	3.419	0.008 *
CD4+TLR-7+ [%] & CD19+TLR-8+ [%]	0.755	3.450	0.007 *
CD8+TLR-2+ [%] & CD4+TLR-8+ [%]	0.764	3.548	0.006 *
CD4+TLR-8+ [%] & CD8+TLR-2+ [%]	0.764	3.548	0.006 *
sTLR-2 in serum [ng/mL] & CD8+TLR-9+ [%]	0.764	3.548	0.006 *
CD8+TLR-3+ [%] & CD19+TLR-3+ [%]	0.781	3.749	0.005 *
CD19+TLR-2+ [%] & sTLR-2 in serum [ng/mL]	0.782	3.762	0.004 *
sTLR-2 in serum [ng/mL] & CD19+TLR-2+ [%]	0.782	3.762	0.004 *
CD4+TLR-2+ [%] & CD8+TLR-8+ [%]	0.783	3.772	0.004 *
CD8+TLR-4+ [%] & CD19+TLR-9+ [%]	0.791	3.877	0.004 *
CD19+TLR-9+ [%] & CD8+TLR-4+ [%]	0.791	3.877	0.004 *
CD4+TLR-8+ [%] & CD19+TLR-8+ [%]	0.795	3.925	0.003 *
CD8+TLR-2+ [%] & CD8+TLR-7+ [%]	0.800	4.000	0.003 *
CD8+TLR-4+ [%] & sTLR-4 in serum [ng/mL]	0.800	4.000	0.003 *
CD4+TLR-9+ [%] & CD19+TLR-2+ [%]	0.800	4.000	0.003 *
CD19+TLR-9+ [%] & CD8+TLR-9+ [%]	0.800	4.000	0.003 *
CD19+TLR-2+ [%] & sTLR-4 in serum [ng/mL]	0.809	4.130	0.003 *
CD4+TLR-9+ [%] & CD8+TLR-4+ [%]	0.809	4.130	0.003 *
sTLR-4 in serum [ng/mL] & CD19+TLR-2+ [%]	0.809	4.130	0.003 *
CD4+TLR-2+ [%] & CD8+TLR-3+ [%]	0.811	4.158	0.002 *
sTLR-4 in serum [ng/mL] & sTLR-9 in serum [ng/mL]	0.818	4.269	0.002 *
CD8+TLR-3+ [%] & CD19+TLR-8+ [%]	0.819	4.286	0.002 *
CD19+TLR-2+ [%] & CD4+TLR-7+ [%]	0.822	4.329	0.002 *
CD19+TLR-2+ [%] & CD8+TLR-4+ [%]	0.827	4.418	0.002 *
CD19+TLR-3+ [%] & CD4+TLR-7+ [%]	0.828	4.436	0.002 *

CD19+TLR-8+ [%] & sTLR-3 in serum [ng/mL]	0.831	4.483	0.002 *
CD4+TLR-4+ [%] & CD8+TLR-4+ [%]	0.836	4.577	0.001 *
CD19+TLR-3+ [%] & CD8+TLR-8+ [%]	0.844	4.722	0.001 *
CD4+TLR-9+ [%] & sTLR-4 in serum [ng/mL]	0.845	4.749	0.001 *
CD8+TLR-3+ [%] & CD8+TLR-7+ [%]	0.852	4.881	0.001 *
CD8+TLR-7+ [%] & CD4+TLR-8+ [%]	0.855	4.936	0.001 *
CD19+TLR-9+ [%] & sTLR-2 in serum [ng/mL]	0.855	4.936	0.001 *
sTLR-2 in serum [ng/mL] & CD19+TLR-9+ [%]	0.855	4.936	0.001 *
CD19+TLR-3+ [%] & sTLR-3 in serum [ng/mL]	0.861	5.080	0.001 *
CD19+TLR-2+ [%] & CD19+TLR-9+ [%]	0.864	5.140	0.001 *
CD8+TLR-4+ [%] & CD8+TLR-9+ [%]	0.873	5.363	0.000 *
CD8+TLR-8+ [%] & CD19+TLR-8+ [%]	0.880	5.571	0.000 *
CD4+TLR-2+ [%] & CD8+TLR-7+ [%]	0.882	5.610	0.000 *
CD19+TLR-7+ [%] & CD19+TLR-8+ [%]	0.886	5.728	0.000 *
sTLR-2 in serum [ng/mL] & sTLR-4 in serum [ng/mL]	0.891	5.885	0.000 *
sTLR-4 in serum [ng/mL] & sTLR-2 in serum [ng/mL]	0.891	5.885	0.000 *
CD19+TLR-3+ [%] & CD19+TLR-7+ [%]	0.897	6.105	0.000 *
CD4+TLR-2+ [%] & CD4+TLR-8+ [%]	0.900	6.194	0.000 *
CD4+TLR-8+ [%] & CD4+TLR-2+ [%]	0.900	6.194	0.000 *
CD4+TLR-9+ [%] & CD19+TLR-9+ [%]	0.900	6.194	0.000 *
CD19+TLR-9+ [%] & CD4+TLR-9+ [%]	0.900	6.194	0.000 *
CD4+TLR-9+ [%] & sTLR-2 in serum [ng/mL]	0.918	6.953	0.000 *
CD4+TLR-9+ [%] & sTLR-9 in serum [ng/mL]	0.936	8.002	0.000 *
CD4+TLR-8+ [%] & CD8+TLR-8+ [%]	0.938	8.135	0.000 *
CD4+TLR-7+ [%] & CD19+TLR-7+ [%]	0.941	8.315	0.000 *
CD8+TLR-3+ [%] & CD4+TLR-8+ [%]	0.943	8.505	0.000 *
CD19+TLR-3+ [%] & CD19+TLR-8+ [%]	0.952	9.325	0.000 *
CD19+TLR-9+ [%] & sTLR-9 in serum [ng/mL]	0.955	9.607	0.000 *
CD8+TLR-3+ [%] & CD8+TLR-8+ [%]	0.959	10.116	0.000 *
CD4+TLR-7+ [%] & sTLR-3 in serum [ng/mL]	0.968	11.581	0.000 *
CD19+TLR-7+ [%] & sTLR-3 in serum [ng/mL]	0.991	22.097	0.000 *

Table S8. Spearman's rank correlation analysis for patients who did not survive the 3-year follow-up period

Para zmiennych	R	t(N-2)	p
CD8+TLR-3+ [%] & CD19+TLR-4+ [%]	0.307	2.373	0.021*
CD19+TLR-4+ [%] & CD4+TLR-8+ [%]	0.368	2.910	0.005*
CD19+TLR-4+ [%] & sTLR-9 in serum [ng/mL]	0.377	2.989	0.004*
CD19+TLR-4+ [%] & CD19+TLR-9+ [%]	0.398	3.191	0.002*
CD19+TLR-4+ [%] & CD19+TLR-8+ [%]	0.400	3.210	0.002*
CD4+TLR-9+ [%] & sTLR-7 in serum [ng/mL]	0.416	3.359	0.001*
CD19+TLR-4+ [%] & CD4+TLR-9+ [%]	0.437	3.571	0.001*
CD8+TLR-3+ [%] & CD4+TLR-9+ [%]	0.442	3.622	0.001*
CD19+TLR-4+ [%] & CD19+TLR-7+ [%]	0.443	3.634	0.001*
CD8+TLR-7+ [%] & CD4+TLR-9+ [%]	0.444	3.646	0.001*
CD19+TLR-4+ [%] & CD4+TLR-7+ [%]	0.448	3.687	0.001*
CD19+TLR-4+ [%] & CD8+TLR-9+ [%]	0.456	3.768	0.000*
CD8+TLR-3+ [%] & CD8+TLR-9+ [%]	0.469	3.901	0.000*
CD8+TLR-3+ [%] & CD4+TLR-4+ [%]	0.473	3.949	0.000*
CD19+TLR-3+ [%] & CD19+TLR-4+ [%]	0.478	4.001	0.000*

CD4+TLR-8+ [%] & CD4+TLR-9+ [%]	0.479	4.010	0.000*
CD8+TLR-9+ [%] & sTLR-7 in serum [ng/mL]	0.492	4.148	0.000*
CD8+TLR-3+ [%] & sTLR-2 in serum [ng/mL]	0.493	4.166	0.000*
CD19+TLR-4+ [%] & sTLR-4 in serum [ng/mL]	0.494	4.176	0.000*
CD8+TLR-3+ [%] & sTLR-9 in serum [ng/mL]	0.495	4.190	0.000*
CD8+TLR-7+ [%] & sTLR-9 in serum [ng/mL]	0.496	4.198	0.000*
CD8+TLR-2+ [%] & CD19+TLR-8+ [%]	0.507	4.324	0.000*
CD8+TLR-2+ [%] & CD8+TLR-3+ [%]	0.512	4.383	0.000*
CD19+TLR-4+ [%] & CD8+TLR-8+ [%]	0.516	4.427	0.000*
CD19+TLR-4+ [%] & CD8+TLR-7+ [%]	0.517	4.433	0.000*
CD4+TLR-4+ [%] & sTLR-7 in serum [ng/mL]	0.520	4.474	0.000*
CD19+TLR-4+ [%] & sTLR-2 in serum [ng/mL]	0.521	4.485	0.000*
CD8+TLR-3+ [%] & CD19+TLR-9+ [%]	0.522	4.499	0.000*
CD19+TLR-8+ [%] & CD4+TLR-9+ [%]	0.523	4.515	0.000*
CD4+TLR-7+ [%] & CD4+TLR-9+ [%]	0.527	4.555	0.000*
CD8+TLR-7+ [%] & CD8+TLR-9+ [%]	0.534	4.637	0.000*
CD8+TLR-4+ [%] & sTLR-7 in serum [ng/mL]	0.540	4.709	0.000*
CD19+TLR-9+ [%] & sTLR-7 in serum [ng/mL]	0.544	4.762	0.000*
CD4+TLR-2+ [%] & CD4+TLR-9+ [%]	0.544	4.766	0.000*
CD19+TLR-4+ [%] & sTLR-8 in serum [ng/mL]	0.547	4.800	0.000*
CD8+TLR-3+ [%] & CD8+TLR-4+ [%]	0.548	4.811	0.000*
CD19+TLR-2+ [%] & CD19+TLR-4+ [%]	0.549	4.827	0.000*
CD19+TLR-8+ [%] & CD19+TLR-9+ [%]	0.551	4.850	0.000*
CD19+TLR-2+ [%] & CD8+TLR-3+ [%]	0.555	4.903	0.000*
CD19+TLR-8+ [%] & CD8+TLR-9+ [%]	0.560	4.967	0.000*
CD19+TLR-3+ [%] & CD4+TLR-9+ [%]	0.561	4.983	0.000*
CD19+TLR-7+ [%] & CD4+TLR-9+ [%]	0.562	4.990	0.000*
sTLR-7 in serum [ng/mL] & sTLR-9 in serum [ng/mL]	0.562	4.999	0.000*
CD4+TLR-8+ [%] & sTLR-2 in serum [ng/mL]	0.563	5.002	0.000*
CD4+TLR-8+ [%] & CD19+TLR-9+ [%]	0.564	5.014	0.000*
CD4+TLR-2+ [%] & CD19+TLR-8+ [%]	0.566	5.039	0.000*

CD4+TLR-8+ [%] & CD8+TLR-9+ [%]	0.567	5.058	0.000*
CD4+TLR-2+ [%] & CD4+TLR-4+ [%]	0.567	5.065	0.000*
CD8+TLR-7+ [%] & CD19+TLR-9+ [%]	0.568	5.066	0.000*
CD4+TLR-4+ [%] & CD19+TLR-8+ [%]	0.569	5.088	0.000*
CD4+TLR-8+ [%] & sTLR-9 in serum [ng/mL]	0.569	5.090	0.000*
CD4+TLR-2+ [%] & CD19+TLR-4+ [%]	0.570	5.092	0.000*
CD8+TLR-2+ [%] & CD4+TLR-7+ [%]	0.570	5.096	0.000*
CD19+TLR-8+ [%] & sTLR-9 in serum [ng/mL]	0.570	5.102	0.000*
CD8+TLR-2+ [%] & CD4+TLR-9+ [%]	0.573	5.133	0.000*
CD4+TLR-4+ [%] & CD4+TLR-8+ [%]	0.573	5.134	0.000*
CD8+TLR-2+ [%] & CD4+TLR-8+ [%]	0.574	5.155	0.000*
CD19+TLR-8+ [%] & sTLR-2 in serum [ng/mL]	0.577	5.194	0.000*
CD4+TLR-2+ [%] & CD8+TLR-3+ [%]	0.579	5.216	0.000*
CD8+TLR-7+ [%] & sTLR-2 in serum [ng/mL]	0.579	5.217	0.000*
CD4+TLR-7+ [%] & sTLR-9 in serum [ng/mL]	0.580	5.228	0.000*
sTLR-2 in serum [ng/mL] & sTLR-8 in serum [ng/mL]	0.581	5.249	0.000*
CD19+TLR-4+ [%] & sTLR-7 in serum [ng/mL]	0.582	5.266	0.000*
CD8+TLR-4+ [%] & CD19+TLR-4+ [%]	0.583	5.267	0.000*
CD8+TLR-4+ [%] & CD4+TLR-8+ [%]	0.586	5.321	0.000*
CD4+TLR-9+ [%] & sTLR-8 in serum [ng/mL]	0.587	5.330	0.000*
CD8+TLR-8+ [%] & CD4+TLR-9+ [%]	0.589	5.355	0.000*
CD8+TLR-4+ [%] & CD19+TLR-8+ [%]	0.590	5.372	0.000*
CD4+TLR-2+ [%] & sTLR-9 in serum [ng/mL]	0.591	5.380	0.000*
CD4+TLR-3+ [%] & sTLR-9 in serum [ng/mL]	0.591	5.386	0.000*
CD4+TLR-4+ [%] & sTLR-8 in serum [ng/mL]	0.594	5.419	0.000*
CD4+TLR-4+ [%] & CD19+TLR-7+ [%]	0.594	5.422	0.000*
CD4+TLR-4+ [%] & CD4+TLR-7+ [%]	0.594	5.424	0.000*
CD4+TLR-4+ [%] & CD8+TLR-7+ [%]	0.594	5.426	0.000*
CD4+TLR-7+ [%] & CD19+TLR-9+ [%]	0.594	5.429	0.000*
CD19+TLR-2+ [%] & sTLR-7 in serum [ng/mL]	0.595	5.437	0.000*
CD4+TLR-7+ [%] & sTLR-2 in serum [ng/mL]	0.603	5.550	0.000*

CD8+TLR-2+ [%] & sTLR-9 in serum [ng/mL]	0.605	5.578	0.000*
CD4+TLR-2+ [%] & CD4+TLR-8+ [%]	0.607	5.616	0.000*
sTLR-2 in serum [ng/mL] & sTLR-7 in serum [ng/mL]	0.609	5.646	0.000*
CD4+TLR-2+ [%] & sTLR-2 in serum [ng/mL]	0.610	5.654	0.000*
CD8+TLR-8+ [%] & sTLR-9 in serum [ng/mL]	0.614	5.713	0.000*
CD8+TLR-4+ [%] & CD8+TLR-7+ [%]	0.615	5.727	0.000*
CD4+TLR-2+ [%] & CD8+TLR-4+ [%]	0.617	5.757	0.000*
CD19+TLR-3+ [%] & CD4+TLR-4+ [%]	0.617	5.763	0.000*
CD4+TLR-3+ [%] & CD19+TLR-4+ [%]	0.618	5.773	0.000*
CD8+TLR-2+ [%] & CD19+TLR-7+ [%]	0.621	5.828	0.000*
CD4+TLR-2+ [%] & CD8+TLR-7+ [%]	0.622	5.835	0.000*
CD19+TLR-7+ [%] & sTLR-9 in serum [ng/mL]	0.622	5.837	0.000*
CD19+TLR-7+ [%] & sTLR-2 in serum [ng/mL]	0.623	5.853	0.000*
CD4+TLR-7+ [%] & CD8+TLR-9+ [%]	0.628	5.936	0.000*
CD19+TLR-3+ [%] & sTLR-9 in serum [ng/mL]	0.629	5.939	0.000*
CD19+TLR-7+ [%] & CD8+TLR-9+ [%]	0.633	6.012	0.000*
CD19+TLR-8+ [%] & sTLR-4 in serum [ng/mL]	0.634	6.031	0.000*
CD8+TLR-4+ [%] & CD4+TLR-7+ [%]	0.636	6.055	0.000*
CD4+TLR-3+ [%] & CD4+TLR-9+ [%]	0.639	6.107	0.000*
CD19+TLR-7+ [%] & CD19+TLR-9+ [%]	0.640	6.114	0.000*
CD19+TLR-4+ [%] & sTLR-3 in serum [ng/mL]	0.640	6.115	0.000*
CD4+TLR-4+ [%] & sTLR-9 in serum [ng/mL]	0.640	6.118	0.000*
CD4+TLR-4+ [%] & CD8+TLR-8+ [%]	0.640	6.118	0.000*
CD4+TLR-7+ [%] & sTLR-4 in serum [ng/mL]	0.641	6.135	0.000*
CD19+TLR-3+ [%] & sTLR-2 in serum [ng/mL]	0.641	6.138	0.000*
CD4+TLR-2+ [%] & CD8+TLR-9+ [%]	0.643	6.163	0.000*
CD19+TLR-2+ [%] & CD4+TLR-8+ [%]	0.645	6.197	0.000*
sTLR-8 in serum [ng/mL] & sTLR-9 in serum [ng/mL]	0.645	6.202	0.000*
CD8+TLR-2+ [%] & CD8+TLR-8+ [%]	0.645	6.209	0.000*
CD8+TLR-2+ [%] & CD19+TLR-3+ [%]	0.646	6.212	0.000*
CD19+TLR-3+ [%] & CD8+TLR-9+ [%]	0.646	6.227	0.000*

CD19+TLR-2+ [%] & CD4+TLR-7+ [%]	0.649	6.269	0.000*
CD4+TLR-8+ [%] & sTLR-4 in serum [ng/mL]	0.651	6.301	0.000*
CD19+TLR-3+ [%] & CD19+TLR-9+ [%]	0.651	6.302	0.000*
sTLR-3 in serum [ng/mL] & sTLR-9 in serum [ng/mL]	0.653	6.337	0.000*
CD4+TLR-3+ [%] & CD19+TLR-9+ [%]	0.653	6.340	0.000*
CD4+TLR-4+ [%] & CD19+TLR-9+ [%]	0.654	6.346	0.000*
CD4+TLR-9+ [%] & sTLR-3 in serum [ng/mL]	0.657	6.412	0.000*
CD4+TLR-2+ [%] & CD4+TLR-7+ [%]	0.660	6.450	0.000*
CD8+TLR-8+ [%] & sTLR-2 in serum [ng/mL]	0.660	6.456	0.000*
CD19+TLR-8+ [%] & sTLR-7 in serum [ng/mL]	0.660	6.459	0.000*
CD19+TLR-2+ [%] & CD19+TLR-8+ [%]	0.662	6.499	0.000*
CD8+TLR-4+ [%] & sTLR-8 in serum [ng/mL]	0.663	6.509	0.000*
CD8+TLR-2+ [%] & CD19+TLR-9+ [%]	0.663	6.510	0.000*
CD8+TLR-2+ [%] & sTLR-7 in serum [ng/mL]	0.665	6.549	0.000*
CD4+TLR-2+ [%] & CD19+TLR-7+ [%]	0.665	6.551	0.000*
CD8+TLR-2+ [%] & CD19+TLR-4+ [%]	0.666	6.561	0.000*
CD8+TLR-8+ [%] & CD8+TLR-9+ [%]	0.666	6.568	0.000*
CD4+TLR-2+ [%] & CD19+TLR-9+ [%]	0.667	6.583	0.000*
CD8+TLR-3+ [%] & CD19+TLR-8+ [%]	0.667	6.583	0.000*
CD8+TLR-4+ [%] & CD8+TLR-8+ [%]	0.670	6.635	0.000*
CD8+TLR-4+ [%] & CD19+TLR-7+ [%]	0.671	6.643	0.000*
CD8+TLR-9+ [%] & sTLR-8 in serum [ng/mL]	0.672	6.665	0.000*
CD8+TLR-2+ [%] & CD8+TLR-9+ [%]	0.673	6.681	0.000*
CD4+TLR-4+ [%] & sTLR-3 in serum [ng/mL]	0.674	6.706	0.000*
CD19+TLR-2+ [%] & sTLR-8 in serum [ng/mL]	0.675	6.714	0.000*
sTLR-4 in serum [ng/mL] & sTLR-9 in serum [ng/mL]	0.675	6.714	0.000*
CD19+TLR-8+ [%] & sTLR-8 in serum [ng/mL]	0.675	6.721	0.000*
CD8+TLR-2+ [%] & sTLR-8 in serum [ng/mL]	0.677	6.760	0.000*
CD8+TLR-3+ [%] & sTLR-4 in serum [ng/mL]	0.680	6.808	0.000*
CD8+TLR-2+ [%] & CD4+TLR-3+ [%]	0.681	6.826	0.000*
CD19+TLR-9+ [%] & sTLR-8 in serum [ng/mL]	0.682	6.847	0.000*

CD19+TLR-2+ [%] & CD19+TLR-7+ [%]	0.682	6.855	0.000*
CD8+TLR-8+ [%] & CD19+TLR-9+ [%]	0.683	6.866	0.000*
CD4+TLR-2+ [%] & CD8+TLR-2+ [%]	0.683	6.876	0.000*
CD4+TLR-2+ [%] & CD19+TLR-3+ [%]	0.683	6.876	0.000*
CD4+TLR-3+ [%] & sTLR-2 in serum [ng/mL]	0.683	6.880	0.000*
CD19+TLR-3+ [%] & CD8+TLR-4+ [%]	0.685	6.918	0.000*
CD8+TLR-2+ [%] & CD8+TLR-7+ [%]	0.687	6.948	0.000*
sTLR-4 in serum [ng/mL] & sTLR-8 in serum [ng/mL]	0.688	6.963	0.000*
CD4+TLR-3+ [%] & CD8+TLR-3+ [%]	0.688	6.972	0.000*
CD4+TLR-3+ [%] & CD4+TLR-4+ [%]	0.691	7.016	0.000*
CD8+TLR-3+ [%] & sTLR-8 in serum [ng/mL]	0.691	7.032	0.000*
CD4+TLR-3+ [%] & CD8+TLR-4+ [%]	0.696	7.128	0.000*
CD8+TLR-2+ [%] & sTLR-2 in serum [ng/mL]	0.696	7.131	0.000*
CD8+TLR-7+ [%] & CD19+TLR-8+ [%]	0.703	7.259	0.000*
sTLR-2 in serum [ng/mL] & sTLR-3 in serum [ng/mL]	0.704	7.281	0.000*
CD4+TLR-2+ [%] & CD19+TLR-2+ [%]	0.705	7.302	0.000*
CD19+TLR-2+ [%] & sTLR-9 in serum [ng/mL]	0.706	7.317	0.000*
CD4+TLR-4+ [%] & CD19+TLR-4+ [%]	0.706	7.326	0.000*
CD4+TLR-2+ [%] & CD4+TLR-3+ [%]	0.707	7.349	0.000*
CD19+TLR-2+ [%] & CD8+TLR-7+ [%]	0.708	7.358	0.000*
CD8+TLR-3+ [%] & sTLR-3 in serum [ng/mL]	0.708	7.377	0.000*
CD8+TLR-3+ [%] & sTLR-7 in serum [ng/mL]	0.709	7.379	0.000*
CD8+TLR-2+ [%] & CD8+TLR-4+ [%]	0.709	7.387	0.000*
CD8+TLR-3+ [%] & CD8+TLR-8+ [%]	0.709	7.390	0.000*
CD4+TLR-2+ [%] & sTLR-8 in serum [ng/mL]	0.715	7.518	0.000*
CD19+TLR-2+ [%] & CD19+TLR-3+ [%]	0.719	7.611	0.000*
CD4+TLR-2+ [%] & sTLR-4 in serum [ng/mL]	0.722	7.671	0.000*
CD8+TLR-2+ [%] & CD4+TLR-4+ [%]	0.723	7.685	0.000*
CD19+TLR-7+ [%] & sTLR-4 in serum [ng/mL]	0.724	7.704	0.000*
CD4+TLR-4+ [%] & sTLR-4 in serum [ng/mL]	0.725	7.727	0.000*
CD8+TLR-2+ [%] & sTLR-4 in serum [ng/mL]	0.725	7.737	0.000*

sTLR-4 in serum [ng/mL] & sTLR-7 in serum [ng/mL]	0.728	7.798	0.000*
CD4+TLR-4+ [%] & CD8+TLR-9+ [%]	0.728	7.799	0.000*
CD4+TLR-9+ [%] & sTLR-4 in serum [ng/mL]	0.728	7.806	0.000*
CD19+TLR-9+ [%] & sTLR-3 in serum [ng/mL]	0.728	7.809	0.000*
CD4+TLR-3+ [%] & CD8+TLR-9+ [%]	0.730	7.838	0.000*
CD19+TLR-3+ [%] & sTLR-4 in serum [ng/mL]	0.732	7.889	0.000*
CD19+TLR-8+ [%] & sTLR-3 in serum [ng/mL]	0.734	7.934	0.000*
CD19+TLR-2+ [%] & CD4+TLR-9+ [%]	0.738	8.046	0.000*
CD4+TLR-3+ [%] & sTLR-7 in serum [ng/mL]	0.740	8.090	0.000*
CD4+TLR-2+ [%] & CD8+TLR-8+ [%]	0.741	8.102	0.000*
CD8+TLR-4+ [%] & sTLR-3 in serum [ng/mL]	0.741	8.108	0.000*
CD8+TLR-3+ [%] & CD8+TLR-7+ [%]	0.745	8.211	0.000*
CD8+TLR-9+ [%] & sTLR-4 in serum [ng/mL]	0.749	8.306	0.000*
CD8+TLR-2+ [%] & sTLR-3 in serum [ng/mL]	0.750	8.332	0.000*
CD4+TLR-3+ [%] & CD19+TLR-8+ [%]	0.753	8.404	0.000*
CD8+TLR-7+ [%] & sTLR-4 in serum [ng/mL]	0.754	8.441	0.000*
CD4+TLR-7+ [%] & CD8+TLR-7+ [%]	0.755	8.468	0.000*
CD8+TLR-9+ [%] & sTLR-3 in serum [ng/mL]	0.756	8.483	0.000*
CD4+TLR-2+ [%] & sTLR-7 in serum [ng/mL]	0.758	8.534	0.000*
CD19+TLR-2+ [%] & CD4+TLR-3+ [%]	0.758	8.540	0.000*
CD4+TLR-9+ [%] & sTLR-9 in serum [ng/mL]	0.761	8.623	0.000*
CD8+TLR-9+ [%] & sTLR-9 in serum [ng/mL]	0.762	8.642	0.000*
CD4+TLR-3+ [%] & CD8+TLR-7+ [%]	0.766	8.764	0.000*
CD8+TLR-8+ [%] & sTLR-8 in serum [ng/mL]	0.767	8.789	0.000*
CD19+TLR-7+ [%] & CD19+TLR-8+ [%]	0.769	8.836	0.000*
CD8+TLR-2+ [%] & CD19+TLR-2+ [%]	0.769	8.846	0.000*
CD19+TLR-2+ [%] & CD19+TLR-9+ [%]	0.772	8.938	0.000*
CD8+TLR-8+ [%] & sTLR-7 in serum [ng/mL]	0.773	8.948	0.000*
CD4+TLR-3+ [%] & CD4+TLR-8+ [%]	0.774	8.972	0.000*
CD8+TLR-4+ [%] & sTLR-4 in serum [ng/mL]	0.775	9.010	0.000*
CD4+TLR-4+ [%] & sTLR-2 in serum [ng/mL]	0.776	9.027	0.000*

sTLR-3 in serum [ng/mL] & sTLR-4 in serum [ng/mL]	0.776	9.029	0.000*
CD4+TLR-4+ [%] & CD4+TLR-9+ [%]	0.776	9.033	0.000*
CD4+TLR-7+ [%] & CD19+TLR-8+ [%]	0.777	9.078	0.000*
CD4+TLR-3+ [%] & sTLR-4 in serum [ng/mL]	0.779	9.126	0.000*
CD4+TLR-8+ [%] & sTLR-7 in serum [ng/mL]	0.779	9.137	0.000*
CD8+TLR-7+ [%] & sTLR-8 in serum [ng/mL]	0.780	9.147	0.000*
CD19+TLR-9+ [%] & sTLR-4 in serum [ng/mL]	0.782	9.229	0.000*
CD4+TLR-7+ [%] & sTLR-7 in serum [ng/mL]	0.783	9.263	0.000*
CD19+TLR-2+ [%] & CD8+TLR-8+ [%]	0.784	9.268	0.000*
sTLR-7 in serum [ng/mL] & sTLR-8 in serum [ng/mL]	0.784	9.295	0.000*
CD19+TLR-2+ [%] & sTLR-3 in serum [ng/mL]	0.786	9.354	0.000*
CD4+TLR-8+ [%] & sTLR-3 in serum [ng/mL]	0.788	9.406	0.000*
CD8+TLR-8+ [%] & sTLR-4 in serum [ng/mL]	0.789	9.426	0.000*
sTLR-2 in serum [ng/mL] & sTLR-9 in serum [ng/mL]	0.790	9.476	0.000*
CD19+TLR-2+ [%] & CD8+TLR-9+ [%]	0.795	9.616	0.000*
CD19+TLR-2+ [%] & CD4+TLR-4+ [%]	0.795	9.632	0.000*
CD4+TLR-3+ [%] & sTLR-8 in serum [ng/mL]	0.797	9.688	0.000*
CD8+TLR-7+ [%] & sTLR-7 in serum [ng/mL]	0.799	9.753	0.000*
CD8+TLR-7+ [%] & CD8+TLR-8+ [%]	0.801	9.828	0.000*
CD4+TLR-7+ [%] & CD8+TLR-8+ [%]	0.802	9.872	0.000*
CD19+TLR-9+ [%] & sTLR-9 in serum [ng/mL]	0.802	9.880	0.000*
CD4+TLR-3+ [%] & CD8+TLR-8+ [%]	0.803	9.884	0.000*
CD8+TLR-4+ [%] & sTLR-9 in serum [ng/mL]	0.803	9.917	0.000*
CD4+TLR-8+ [%] & sTLR-8 in serum [ng/mL]	0.803	9.917	0.000*
CD8+TLR-7+ [%] & CD4+TLR-8+ [%]	0.804	9.935	0.000*
CD4+TLR-3+ [%] & CD4+TLR-7+ [%]	0.806	9.996	0.000*
CD19+TLR-3+ [%] & CD19+TLR-8+ [%]	0.809	10.098	0.000*
CD8+TLR-3+ [%] & CD4+TLR-7+ [%]	0.809	10.099	0.000*
CD4+TLR-7+ [%] & sTLR-8 in serum [ng/mL]	0.811	10.189	0.000*
CD19+TLR-7+ [%] & CD8+TLR-8+ [%]	0.813	10.268	0.000*
CD4+TLR-3+ [%] & CD19+TLR-7+ [%]	0.816	10.363	0.000*

CD19+TLR-2+ [%] & sTLR-2 in serum [ng/mL]	0.818	10.458	0.000*
CD4+TLR-8+ [%] & CD19+TLR-8+ [%]	0.819	10.480	0.000*
CD8+TLR-9+ [%] & sTLR-2 in serum [ng/mL]	0.822	10.592	0.000*
CD4+TLR-2+ [%] & sTLR-3 in serum [ng/mL]	0.822	10.610	0.000*
CD4+TLR-3+ [%] & CD19+TLR-3+ [%]	0.825	10.726	0.000*
CD19+TLR-2+ [%] & CD8+TLR-4+ [%]	0.825	10.736	0.000*
CD4+TLR-9+ [%] & sTLR-2 in serum [ng/mL]	0.826	10.773	0.000*
CD19+TLR-9+ [%] & sTLR-2 in serum [ng/mL]	0.827	10.801	0.000*
CD8+TLR-7+ [%] & sTLR-3 in serum [ng/mL]	0.827	10.802	0.000*
CD8+TLR-4+ [%] & CD19+TLR-9+ [%]	0.830	10.929	0.000*
CD8+TLR-4+ [%] & CD8+TLR-9+ [%]	0.830	10.932	0.000*
sTLR-2 in serum [ng/mL] & sTLR-4 in serum [ng/mL]	0.830	10.937	0.000*
CD8+TLR-7+ [%] & CD19+TLR-7+ [%]	0.830	10.947	0.000*
CD4+TLR-8+ [%] & CD8+TLR-8+ [%]	0.831	10.991	0.000*
CD19+TLR-7+ [%] & sTLR-7 in serum [ng/mL]	0.833	11.066	0.000*
CD4+TLR-4+ [%] & CD8+TLR-4+ [%]	0.839	11.330	0.000*
CD8+TLR-3+ [%] & CD4+TLR-8+ [%]	0.841	11.446	0.000*
CD19+TLR-3+ [%] & sTLR-7 in serum [ng/mL]	0.842	11.457	0.000*
CD8+TLR-8+ [%] & sTLR-3 in serum [ng/mL]	0.843	11.536	0.000*
CD4+TLR-7+ [%] & sTLR-3 in serum [ng/mL]	0.848	11.748	0.000*
CD8+TLR-3+ [%] & CD19+TLR-3+ [%]	0.850	11.869	0.000*
sTLR-3 in serum [ng/mL] & sTLR-7 in serum [ng/mL]	0.853	12.002	0.000*
CD19+TLR-3+ [%] & CD8+TLR-8+ [%]	0.856	12.192	0.000*
CD19+TLR-7+ [%] & sTLR-8 in serum [ng/mL]	0.858	12.260	0.000*
CD19+TLR-2+ [%] & sTLR-4 in serum [ng/mL]	0.859	12.304	0.000*
CD8+TLR-4+ [%] & sTLR-2 in serum [ng/mL]	0.859	12.306	0.000*
CD19+TLR-3+ [%] & CD8+TLR-7+ [%]	0.861	12.444	0.000*
CD8+TLR-8+ [%] & CD19+TLR-8+ [%]	0.863	12.540	0.000*
CD4+TLR-9+ [%] & CD19+TLR-9+ [%]	0.864	12.587	0.000*
CD8+TLR-3+ [%] & CD19+TLR-7+ [%]	0.867	12.775	0.000*
CD19+TLR-3+ [%] & sTLR-8 in serum [ng/mL]	0.867	12.777	0.000*

CD4+TLR-9+ [%] & CD8+TLR-9+ [%]	0.871	12.999	0.000*
sTLR-3 in serum [ng/mL] & sTLR-8 in serum [ng/mL]	0.873	13.154	0.000*
CD19+TLR-7+ [%] & sTLR-3 in serum [ng/mL]	0.874	13.200	0.000*
CD8+TLR-4+ [%] & CD4+TLR-9+ [%]	0.883	13.793	0.000*
CD19+TLR-3+ [%] & sTLR-3 in serum [ng/mL]	0.888	14.199	0.000*
CD4+TLR-3+ [%] & sTLR-3 in serum [ng/mL]	0.897	14.943	0.000*
CD8+TLR-9+ [%] & CD19+TLR-9+ [%]	0.909	16.074	0.000*
CD4+TLR-7+ [%] & CD4+TLR-8+ [%]	0.932	18.962	0.000*
CD4+TLR-7+ [%] & CD19+TLR-7+ [%]	0.939	20.118	0.000*
CD19+TLR-3+ [%] & CD4+TLR-7+ [%]	0.940	20.300	0.000*
CD19+TLR-7+ [%] & CD4+TLR-8+ [%]	0.955	23.598	0.000*
CD19+TLR-3+ [%] & CD4+TLR-8+ [%]	0.960	25.038	0.000*
CD19+TLR-3+ [%] & CD19+TLR-7+ [%]	0.988	46.873	0.000*

Table S9. Spearman's rank correlation analysis for stage IV patients at the time of recruitment and study

Para zmiennych	R	t(N-2)	p
CD19+TLR-4+ [%] & sTLR-9 in serum [ng/mL]	0.399	2.088	0.048*
CD19+TLR-8+ [%] & CD4+TLR-9+ [%]	0.401	2.099	0.047*
CD8+TLR-8+ [%] & CD4+TLR-9+ [%]	0.407	2.139	0.043*
CD8+TLR-4+ [%] & CD8+TLR-8+ [%]	0.421	2.225	0.036*
CD4+TLR-4+ [%] & sTLR-9 in serum [ng/mL]	0.421	2.228	0.036*
CD4+TLR-4+ [%] & CD19+TLR-8+ [%]	0.424	2.244	0.035*
sTLR-4 in serum [ng/mL] & sTLR-8 in serum [ng/mL]	0.424	2.245	0.035*
CD19+TLR-4+ [%] & sTLR-4 in serum [ng/mL]	0.425	2.254	0.034*
CD4+TLR-3+ [%] & CD4+TLR-9+ [%]	0.430	2.287	0.032*
CD19+TLR-8+ [%] & CD8+TLR-9+ [%]	0.431	2.291	0.031*
CD19+TLR-7+ [%] & CD4+TLR-9+ [%]	0.432	2.296	0.031*
CD8+TLR-7+ [%] & CD4+TLR-9+ [%]	0.433	2.301	0.031*
CD19+TLR-3+ [%] & CD4+TLR-9+ [%]	0.433	2.306	0.030*
CD4+TLR-2+ [%] & sTLR-2 in serum [ng/mL]	0.437	2.333	0.029*
CD19+TLR-2+ [%] & CD8+TLR-9+ [%]	0.439	2.345	0.028*
CD4+TLR-7+ [%] & sTLR-2 in serum [ng/mL]	0.441	2.353	0.028*
sTLR-7 in serum [ng/mL] & sTLR-8 in serum [ng/mL]	0.441	2.355	0.027*
CD4+TLR-8+ [%] & sTLR-2 in serum [ng/mL]	0.441	2.356	0.027*
CD4+TLR-9+ [%] & sTLR-3 in serum [ng/mL]	0.442	2.360	0.027*
CD8+TLR-2+ [%] & CD4+TLR-9+ [%]	0.442	2.361	0.027*
CD19+TLR-2+ [%] & sTLR-8 in serum [ng/mL]	0.443	2.369	0.027*
CD19+TLR-4+ [%] & sTLR-7 in serum [ng/mL]	0.448	2.405	0.025*
CD4+TLR-9+ [%] & sTLR-7 in serum [ng/mL]	0.451	2.421	0.024*
CD19+TLR-8+ [%] & sTLR-4 in serum [ng/mL]	0.452	2.429	0.023*
CD4+TLR-2+ [%] & CD4+TLR-4+ [%]	0.462	2.500	0.020*
CD4+TLR-2+ [%] & sTLR-9 in serum [ng/mL]	0.467	2.536	0.018*
CD8+TLR-8+ [%] & CD19+TLR-9+ [%]	0.468	2.538	0.018*
CD19+TLR-8+ [%] & sTLR-9 in serum [ng/mL]	0.470	2.556	0.018*
CD19+TLR-4+ [%] & CD4+TLR-9+ [%]	0.472	2.570	0.017*
CD8+TLR-8+ [%] & sTLR-8 in serum [ng/mL]	0.475	2.588	0.016*
CD19+TLR-8+ [%] & sTLR-2 in serum [ng/mL]	0.482	2.635	0.015*
CD4+TLR-4+ [%] & CD8+TLR-9+ [%]	0.485	2.661	0.014*
CD19+TLR-4+ [%] & CD8+TLR-7+ [%]	0.487	2.673	0.014*
CD4+TLR-3+ [%] & CD19+TLR-4+ [%]	0.489	2.685	0.013*
CD4+TLR-3+ [%] & CD8+TLR-4+ [%]	0.489	2.688	0.013*
CD4+TLR-7+ [%] & sTLR-4 in serum [ng/mL]	0.491	2.700	0.013*
CD8+TLR-2+ [%] & CD19+TLR-4+ [%]	0.492	2.713	0.012*
CD19+TLR-4+ [%] & CD19+TLR-7+ [%]	0.493	2.720	0.012*
CD19+TLR-3+ [%] & CD19+TLR-4+ [%]	0.494	2.723	0.012*
CD8+TLR-2+ [%] & CD19+TLR-9+ [%]	0.495	2.730	0.012*
CD8+TLR-2+ [%] & CD8+TLR-4+ [%]	0.497	2.744	0.012*
CD8+TLR-8+ [%] & sTLR-2 in serum [ng/mL]	0.497	2.745	0.012*
CD4+TLR-8+ [%] & sTLR-4 in serum [ng/mL]	0.497	2.747	0.011*

CD19+TLR-4+ [%] & sTLR-3 in serum [ng/mL]	0.498	2.752	0.011*
CD19+TLR-2+ [%] & CD8+TLR-4+ [%]	0.502	2.780	0.011*
CD19+TLR-2+ [%] & sTLR-9 in serum [ng/mL]	0.505	2.809	0.010*
CD8+TLR-4+ [%] & sTLR-7 in serum [ng/mL]	0.512	2.857	0.009*
CD8+TLR-4+ [%] & CD8+TLR-7+ [%]	0.513	2.869	0.009*
CD8+TLR-2+ [%] & CD19+TLR-8+ [%]	0.520	2.921	0.008*
CD8+TLR-4+ [%] & CD19+TLR-7+ [%]	0.521	2.928	0.008*
CD8+TLR-8+ [%] & sTLR-9 in serum [ng/mL]	0.521	2.929	0.008*
CD4+TLR-2+ [%] & CD19+TLR-9+ [%]	0.524	2.947	0.007*
CD19+TLR-3+ [%] & CD8+TLR-4+ [%]	0.525	2.955	0.007*
CD4+TLR-3+ [%] & sTLR-8 in serum [ng/mL]	0.525	2.958	0.007*
CD19+TLR-2+ [%] & sTLR-2 in serum [ng/mL]	0.527	2.977	0.007*
CD8+TLR-4+ [%] & sTLR-3 in serum [ng/mL]	0.528	2.979	0.007*
CD4+TLR-2+ [%] & CD19+TLR-8+ [%]	0.529	2.992	0.007*
CD8+TLR-2+ [%] & sTLR-8 in serum [ng/mL]	0.530	2.995	0.006*
CD8+TLR-7+ [%] & CD19+TLR-9+ [%]	0.530	3.000	0.006*
CD8+TLR-7+ [%] & sTLR-8 in serum [ng/mL]	0.531	3.009	0.006*
CD19+TLR-3+ [%] & sTLR-8 in serum [ng/mL]	0.533	3.024	0.006*
CD19+TLR-4+ [%] & sTLR-2 in serum [ng/mL]	0.534	3.026	0.006*
CD4+TLR-3+ [%] & CD19+TLR-9+ [%]	0.534	3.032	0.006*
CD19+TLR-7+ [%] & sTLR-8 in serum [ng/mL]	0.535	3.036	0.006*
sTLR-3 in serum [ng/mL] & sTLR-8 in serum [ng/mL]	0.537	3.054	0.006*
CD8+TLR-8+ [%] & CD19+TLR-8+ [%]	0.538	3.062	0.006*
CD19+TLR-2+ [%] & CD19+TLR-8+ [%]	0.541	3.086	0.005*
CD19+TLR-7+ [%] & CD19+TLR-9+ [%]	0.545	3.114	0.005*
CD4+TLR-4+ [%] & CD4+TLR-9+ [%]	0.553	3.179	0.004*
CD4+TLR-4+ [%] & sTLR-2 in serum [ng/mL]	0.553	3.180	0.004*
CD19+TLR-3+ [%] & CD19+TLR-9+ [%]	0.553	3.180	0.004*
CD8+TLR-2+ [%] & sTLR-9 in serum [ng/mL]	0.553	3.181	0.004*
CD4+TLR-4+ [%] & CD8+TLR-8+ [%]	0.553	3.184	0.004*
CD19+TLR-9+ [%] & sTLR-3 in serum [ng/mL]	0.554	3.190	0.004*
CD4+TLR-4+ [%] & sTLR-7 in serum [ng/mL]	0.555	3.197	0.004*
CD4+TLR-7+ [%] & CD8+TLR-9+ [%]	0.568	3.313	0.003*
CD4+TLR-8+ [%] & CD8+TLR-9+ [%]	0.571	3.335	0.003*
CD8+TLR-4+ [%] & CD19+TLR-4+ [%]	0.577	3.387	0.003*
CD8+TLR-2+ [%] & CD8+TLR-9+ [%]	0.584	3.446	0.002*
CD19+TLR-9+ [%] & sTLR-7 in serum [ng/mL]	0.584	3.452	0.002*
CD8+TLR-2+ [%] & sTLR-2 in serum [ng/mL]	0.584	3.454	0.002*
CD8+TLR-4+ [%] & CD8+TLR-9+ [%]	0.585	3.456	0.002*
CD4+TLR-3+ [%] & sTLR-9 in serum [ng/mL]	0.587	3.474	0.002*
CD8+TLR-8+ [%] & CD8+TLR-9+ [%]	0.590	3.503	0.002*
sTLR-2 in serum [ng/mL] & sTLR-7 in serum [ng/mL]	0.593	3.534	0.002*
CD19+TLR-2+ [%] & CD4+TLR-4+ [%]	0.594	3.545	0.002*
CD4+TLR-4+ [%] & CD4+TLR-7+ [%]	0.596	3.556	0.002*
CD4+TLR-3+ [%] & sTLR-2 in serum [ng/mL]	0.597	3.567	0.002*
CD19+TLR-2+ [%] & CD4+TLR-7+ [%]	0.598	3.575	0.002*
CD19+TLR-9+ [%] & sTLR-4 in serum [ng/mL]	0.598	3.575	0.002*
CD8+TLR-7+ [%] & sTLR-9 in serum [ng/mL]	0.598	3.582	0.002*
CD19+TLR-2+ [%] & CD4+TLR-8+ [%]	0.599	3.584	0.002*
CD4+TLR-4+ [%] & CD4+TLR-8+ [%]	0.599	3.589	0.002*
CD8+TLR-7+ [%] & sTLR-2 in serum [ng/mL]	0.601	3.608	0.001*
CD4+TLR-4+ [%] & CD19+TLR-7+ [%]	0.603	3.624	0.001*
CD19+TLR-7+ [%] & sTLR-2 in serum [ng/mL]	0.603	3.627	0.001*
CD4+TLR-4+ [%] & CD8+TLR-7+ [%]	0.604	3.631	0.001*
CD4+TLR-4+ [%] & sTLR-3 in serum [ng/mL]	0.604	3.635	0.001*
CD19+TLR-7+ [%] & sTLR-9 in serum [ng/mL]	0.604	3.636	0.001*
CD4+TLR-4+ [%] & sTLR-4 in serum [ng/mL]	0.604	3.639	0.001*
CD8+TLR-4+ [%] & CD19+TLR-9+ [%]	0.605	3.648	0.001*
sTLR-7 in serum [ng/mL] & sTLR-9 in serum [ng/mL]	0.606	3.655	0.001*
CD4+TLR-2+ [%] & CD8+TLR-9+ [%]	0.607	3.663	0.001*
CD19+TLR-3+ [%] & CD4+TLR-4+ [%]	0.610	3.692	0.001*
CD19+TLR-3+ [%] & sTLR-9 in serum [ng/mL]	0.610	3.695	0.001*

CD19+TLR-3+ [%] & sTLR-2 in serum [ng/mL]	0.611	3.700	0.001*
sTLR-2 in serum [ng/mL] & sTLR-3 in serum [ng/mL]	0.614	3.734	0.001*
CD4+TLR-3+ [%] & CD4+TLR-4+ [%]	0.615	3.738	0.001*
CD4+TLR-4+ [%] & CD8+TLR-4+ [%]	0.615	3.739	0.001*
sTLR-3 in serum [ng/mL] & sTLR-9 in serum [ng/mL]	0.616	3.752	0.001*
CD8+TLR-7+ [%] & CD19+TLR-8+ [%]	0.623	3.822	0.001*
CD4+TLR-2+ [%] & sTLR-4 in serum [ng/mL]	0.624	3.833	0.001*
CD19+TLR-7+ [%] & CD19+TLR-8+ [%]	0.625	3.841	0.001*
CD19+TLR-8+ [%] & sTLR-3 in serum [ng/mL]	0.626	3.851	0.001*
CD19+TLR-3+ [%] & CD19+TLR-8+ [%]	0.634	3.936	0.001*
CD19+TLR-8+ [%] & sTLR-7 in serum [ng/mL]	0.634	3.937	0.001*
CD8+TLR-2+ [%] & CD4+TLR-8+ [%]	0.648	4.084	0.000*
CD8+TLR-2+ [%] & CD4+TLR-7+ [%]	0.650	4.100	0.000*
CD8+TLR-7+ [%] & CD8+TLR-9+ [%]	0.653	4.140	0.000*
CD19+TLR-7+ [%] & CD8+TLR-9+ [%]	0.655	4.153	0.000*
CD8+TLR-4+ [%] & sTLR-4 in serum [ng/mL]	0.655	4.161	0.000*
CD4+TLR-2+ [%] & sTLR-8 in serum [ng/mL]	0.656	4.164	0.000*
sTLR-4 in serum [ng/mL] & sTLR-7 in serum [ng/mL]	0.660	4.210	0.000*
CD4+TLR-2+ [%] & CD19+TLR-2+ [%]	0.661	4.219	0.000*
CD4+TLR-9+ [%] & sTLR-4 in serum [ng/mL]	0.662	4.239	0.000*
CD4+TLR-2+ [%] & CD4+TLR-8+ [%]	0.664	4.260	0.000*
CD8+TLR-9+ [%] & sTLR-7 in serum [ng/mL]	0.666	4.281	0.000*
CD19+TLR-3+ [%] & CD8+TLR-9+ [%]	0.667	4.288	0.000*
CD4+TLR-2+ [%] & CD4+TLR-7+ [%]	0.668	4.304	0.000*
CD8+TLR-2+ [%] & CD4+TLR-4+ [%]	0.668	4.306	0.000*
CD4+TLR-3+ [%] & CD8+TLR-9+ [%]	0.669	4.312	0.000*
CD8+TLR-9+ [%] & sTLR-3 in serum [ng/mL]	0.669	4.319	0.000*
CD4+TLR-9+ [%] & CD19+TLR-9+ [%]	0.678	4.429	0.000*
CD8+TLR-2+ [%] & sTLR-4 in serum [ng/mL]	0.684	4.497	0.000*
CD4+TLR-7+ [%] & CD8+TLR-8+ [%]	0.689	4.558	0.000*
CD4+TLR-9+ [%] & CD8+TLR-9+ [%]	0.691	4.582	0.000*

CD4+TLR-8+ [%] & CD8+TLR-8+ [%]	0.691	4.584	0.000*
CD4+TLR-3+ [%] & CD19+TLR-8+ [%]	0.692	4.596	0.000*
CD8+TLR-9+ [%] & sTLR-4 in serum [ng/mL]	0.692	4.601	0.000*
CD4+TLR-4+ [%] & CD19+TLR-4+ [%]	0.698	4.679	0.000*
CD4+TLR-3+ [%] & sTLR-4 in serum [ng/mL]	0.702	4.733	0.000*
CD8+TLR-8+ [%] & sTLR-4 in serum [ng/mL]	0.712	4.866	0.000*
CD19+TLR-9+ [%] & sTLR-2 in serum [ng/mL]	0.714	4.896	0.000*
CD19+TLR-7+ [%] & sTLR-4 in serum [ng/mL]	0.715	4.911	0.000*
CD19+TLR-3+ [%] & sTLR-4 in serum [ng/mL]	0.721	4.987	0.000*
sTLR-3 in serum [ng/mL] & sTLR-4 in serum [ng/mL]	0.725	5.054	0.000*
CD8+TLR-7+ [%] & sTLR-4 in serum [ng/mL]	0.727	5.077	0.000*
CD8+TLR-9+ [%] & sTLR-2 in serum [ng/mL]	0.731	5.136	0.000*
CD19+TLR-2+ [%] & sTLR-4 in serum [ng/mL]	0.734	5.181	0.000*
CD19+TLR-2+ [%] & CD8+TLR-8+ [%]	0.748	5.405	0.000*
CD4+TLR-7+ [%] & sTLR-7 in serum [ng/mL]	0.755	5.518	0.000*
CD4+TLR-8+ [%] & sTLR-7 in serum [ng/mL]	0.760	5.602	0.000*
CD4+TLR-7+ [%] & CD8+TLR-7+ [%]	0.763	5.669	0.000*
CD8+TLR-7+ [%] & CD4+TLR-8+ [%]	0.765	5.700	0.000*
sTLR-2 in serum [ng/mL] & sTLR-4 in serum [ng/mL]	0.767	5.727	0.000*
CD4+TLR-7+ [%] & CD19+TLR-7+ [%]	0.767	5.728	0.000*
CD4+TLR-7+ [%] & sTLR-3 in serum [ng/mL]	0.767	5.730	0.000*
CD19+TLR-7+ [%] & CD4+TLR-8+ [%]	0.768	5.756	0.000*
CD4+TLR-8+ [%] & sTLR-3 in serum [ng/mL]	0.769	5.769	0.000*
sTLR-4 in serum [ng/mL] & sTLR-9 in serum [ng/mL]	0.774	5.859	0.000*
CD19+TLR-2+ [%] & sTLR-7 in serum [ng/mL]	0.774	5.865	0.000*
CD8+TLR-9+ [%] & sTLR-9 in serum [ng/mL]	0.775	5.889	0.000*
CD19+TLR-3+ [%] & CD4+TLR-7+ [%]	0.776	5.900	0.000*
CD19+TLR-3+ [%] & CD4+TLR-8+ [%]	0.778	5.933	0.000*
CD19+TLR-2+ [%] & CD4+TLR-3+ [%]	0.778	5.936	0.000*
CD19+TLR-2+ [%] & sTLR-3 in serum [ng/mL]	0.793	6.244	0.000*
sTLR-3 in serum [ng/mL] & CD19+TLR-2+ [%]	0.793	6.244	0.000*

CD19+TLR-2+ [%] & CD19+TLR-3+ [%]	0.795	6.295	0.000*
CD19+TLR-2+ [%] & CD19+TLR-7+ [%]	0.799	6.379	0.000*
CD19+TLR-2+ [%] & CD8+TLR-7+ [%]	0.802	6.439	0.000*
CD8+TLR-9+ [%] & CD19+TLR-9+ [%]	0.815	6.755	0.000*
CD4+TLR-2+ [%] & CD8+TLR-2+ [%]	0.824	6.979	0.000*
CD8+TLR-2+ [%] & CD19+TLR-2+ [%]	0.831	7.173	0.000*
CD4+TLR-7+ [%] & CD19+TLR-8+ [%]	0.835	7.288	0.000*
CD4+TLR-3+ [%] & CD4+TLR-7+ [%]	0.836	7.307	0.000*
CD8+TLR-4+ [%] & CD4+TLR-9+ [%]	0.837	7.333	0.000*
CD4+TLR-8+ [%] & CD19+TLR-8+ [%]	0.838	7.352	0.000*
CD4+TLR-3+ [%] & CD4+TLR-8+ [%]	0.838	7.366	0.000*
CD8+TLR-4+ [%] & sTLR-9 in serum [ng/mL]	0.849	7.713	0.000*
CD8+TLR-2+ [%] & CD8+TLR-8+ [%]	0.850	7.726	0.000*
CD4+TLR-9+ [%] & sTLR-2 in serum [ng/mL]	0.859	8.047	0.000*
CD4+TLR-9+ [%] & sTLR-9 in serum [ng/mL]	0.860	8.082	0.000*
CD8+TLR-2+ [%] & sTLR-7 in serum [ng/mL]	0.864	8.216	0.000*
CD4+TLR-2+ [%] & sTLR-7 in serum [ng/mL]	0.866	8.304	0.000*
CD8+TLR-2+ [%] & CD4+TLR-3+ [%]	0.868	8.399	0.000*
CD8+TLR-4+ [%] & sTLR-2 in serum [ng/mL]	0.875	8.675	0.000*
CD8+TLR-2+ [%] & CD19+TLR-3+ [%]	0.881	8.951	0.000*
CD8+TLR-2+ [%] & CD19+TLR-7+ [%]	0.882	8.968	0.000*
CD8+TLR-2+ [%] & sTLR-3 in serum [ng/mL]	0.882	8.998	0.000*
CD4+TLR-2+ [%] & CD4+TLR-3+ [%]	0.887	9.225	0.000*
CD8+TLR-8+ [%] & sTLR-7 in serum [ng/mL]	0.889	9.310	0.000*
CD8+TLR-2+ [%] & CD8+TLR-7+ [%]	0.894	9.546	0.000*
CD4+TLR-2+ [%] & CD19+TLR-7+ [%]	0.894	9.556	0.000*
CD4+TLR-2+ [%] & CD8+TLR-8+ [%]	0.894	9.575	0.000*
CD4+TLR-2+ [%] & CD8+TLR-7+ [%]	0.894	9.577	0.000*
CD4+TLR-2+ [%] & CD19+TLR-3+ [%]	0.895	9.597	0.000*
CD4+TLR-2+ [%] & sTLR-3 in serum [ng/mL]	0.895	9.632	0.000*
CD4+TLR-3+ [%] & CD8+TLR-8+ [%]	0.911	10.624	0.000*
CD19+TLR-3+ [%] & CD8+TLR-8+ [%]	0.919	11.187	0.000*
CD19+TLR-7+ [%] & CD8+TLR-8+ [%]	0.921	11.341	0.000*
CD8+TLR-8+ [%] & sTLR-3 in serum [ng/mL]	0.922	11.455	0.000*
CD8+TLR-7+ [%] & CD8+TLR-8+ [%]	0.934	12.551	0.000*
sTLR-2 in serum [ng/mL] & sTLR-9 in serum [ng/mL]	0.953	15.064	0.000*
CD4+TLR-3+ [%] & sTLR-7 in serum [ng/mL]	0.964	17.482	0.000*
CD8+TLR-7+ [%] & sTLR-7 in serum [ng/mL]	0.966	18.052	0.000*
CD19+TLR-3+ [%] & sTLR-7 in serum [ng/mL]	0.971	19.650	0.000*
sTLR-3 in serum [ng/mL] & sTLR-7 in serum [ng/mL]	0.972	19.726	0.000*
CD19+TLR-7+ [%] & sTLR-7 in serum [ng/mL]	0.975	20.939	0.000*
CD4+TLR-3+ [%] & CD8+TLR-7+ [%]	0.988	31.284	0.000*
CD4+TLR-3+ [%] & CD19+TLR-7+ [%]	0.992	36.617	0.000*
CD4+TLR-3+ [%] & sTLR-3 in serum [ng/mL]	0.992	37.961	0.000*
CD4+TLR-3+ [%] & CD19+TLR-3+ [%]	0.993	41.716	0.000*
CD19+TLR-3+ [%] & CD8+TLR-7+ [%]	0.995	46.000	0.000*
CD8+TLR-7+ [%] & CD19+TLR-7+ [%]	0.996	53.170	0.000*
CD8+TLR-7+ [%] & sTLR-3 in serum [ng/mL]	0.996	55.933	0.000*
CD4+TLR-7+ [%] & CD4+TLR-8+ [%]	0.998	73.563	0.000*
CD19+TLR-3+ [%] & CD19+TLR-7+ [%]	0.999	122.129	0.000*
CD19+TLR-3+ [%] & sTLR-3 in serum [ng/mL]	0.999	141.104	0.000*
sTLR-3 in serum [ng/mL] & CD19+TLR-3+ [%]	0.999	141.104	0.000*

Table S10. Spearman's rank correlation analysis for stage IV patients at death

Para zmiennych	R	t(N-2)	p
CD19+TLR-4+ [%] & sTLR-7 in serum [ng/mL]	0.401	2.098	0.047*
CD8+TLR-3+ [%] & CD19+TLR-8+ [%]	0.404	2.116	0.045*
CD4+TLR-3+ [%] & CD19+TLR-4+ [%]	0.404	2.119	0.045*
CD4+TLR-8+ [%] & CD4+TLR-9+ [%]	0.408	2.140	0.043*
CD4+TLR-9+ [%] & sTLR-8 in serum [ng/mL]	0.409	2.147	0.043*

CD4+TLR-8+ [%] & sTLR-9 in serum [ng/mL]	0.409	2.152	0.042*
CD4+TLR-4+ [%] & CD8+TLR-8+ [%]	0.410	2.155	0.042*
CD19+TLR-2+ [%] & CD19+TLR-4+ [%]	0.412	2.170	0.041*
CD19+TLR-7+ [%] & sTLR-9 in serum [ng/mL]	0.413	2.172	0.040*
CD4+TLR-2+ [%] & CD4+TLR-9+ [%]	0.414	2.179	0.040*
sTLR-7 in serum [ng/mL] & sTLR-9 in serum [ng/mL]	0.417	2.199	0.038*
CD8+TLR-3+ [%] & sTLR-2 in serum [ng/mL]	0.417	2.200	0.038*
CD19+TLR-8+ [%] & CD4+TLR-9+ [%]	0.418	2.208	0.037*
CD8+TLR-3+ [%] & CD8+TLR-7+ [%]	0.420	2.218	0.037*
CD19+TLR-4+ [%] & CD19+TLR-9+ [%]	0.428	2.268	0.033*
CD19+TLR-8+ [%] & sTLR-4 in serum [ng/mL]	0.431	2.290	0.032*
CD19+TLR-3+ [%] & CD4+TLR-9+ [%]	0.431	2.294	0.031*
CD4+TLR-2+ [%] & CD19+TLR-4+ [%]	0.432	2.299	0.031*
CD8+TLR-4+ [%] & sTLR-7 in serum [ng/mL]	0.434	2.310	0.030*
CD8+TLR-4+ [%] & CD19+TLR-8+ [%]	0.434	2.313	0.030*
CD8+TLR-4+ [%] & CD4+TLR-8+ [%]	0.435	2.314	0.030*
CD4+TLR-2+ [%] & CD8+TLR-3+ [%]	0.440	2.353	0.028*
CD8+TLR-7+ [%] & CD4+TLR-9+ [%]	0.447	2.396	0.025*
CD19+TLR-8+ [%] & sTLR-7 in serum [ng/mL]	0.447	2.400	0.025*
CD19+TLR-4+ [%] & CD8+TLR-7+ [%]	0.448	2.404	0.025*
CD8+TLR-3+ [%] & sTLR-3 in serum [ng/mL]	0.450	2.420	0.024*
CD4+TLR-4+ [%] & CD4+TLR-7+ [%]	0.451	2.421	0.024*
CD4+TLR-2+ [%] & sTLR-9 in serum [ng/mL]	0.451	2.423	0.024*
CD4+TLR-4+ [%] & sTLR-3 in serum [ng/mL]	0.451	2.424	0.024*
CD19+TLR-4+ [%] & sTLR-9 in serum [ng/mL]	0.452	2.429	0.023*
CD8+TLR-3+ [%] & sTLR-7 in serum [ng/mL]	0.452	2.433	0.023*
CD8+TLR-4+ [%] & CD19+TLR-7+ [%]	0.459	2.477	0.021*
CD4+TLR-2+ [%] & CD8+TLR-4+ [%]	0.459	2.479	0.021*
CD8+TLR-4+ [%] & sTLR-8 in serum [ng/mL]	0.459	2.481	0.021*
CD8+TLR-2+ [%] & CD19+TLR-3+ [%]	0.460	2.485	0.021*
CD4+TLR-4+ [%] & sTLR-4 in serum [ng/mL]	0.475	2.592	0.016*
CD8+TLR-9+ [%] & sTLR-7 in serum [ng/mL]	0.476	2.595	0.016*
CD19+TLR-3+ [%] & sTLR-9 in serum [ng/mL]	0.481	2.631	0.015*
CD19+TLR-8+ [%] & CD8+TLR-9+ [%]	0.487	2.673	0.014*
CD4+TLR-7+ [%] & CD4+TLR-9+ [%]	0.502	2.785	0.011*
CD8+TLR-2+ [%] & CD4+TLR-7+ [%]	0.504	2.802	0.010*
CD8+TLR-8+ [%] & CD4+TLR-9+ [%]	0.505	2.803	0.010*
CD4+TLR-7+ [%] & sTLR-9 in serum [ng/mL]	0.505	2.805	0.010*
CD8+TLR-7+ [%] & CD19+TLR-8+ [%]	0.506	2.813	0.010*
CD4+TLR-3+ [%] & CD8+TLR-3+ [%]	0.508	2.826	0.010*
CD19+TLR-8+ [%] & sTLR-9 in serum [ng/mL]	0.509	2.833	0.009*
CD19+TLR-4+ [%] & sTLR-3 in serum [ng/mL]	0.511	2.849	0.009*
CD19+TLR-4+ [%] & CD8+TLR-9+ [%]	0.512	2.861	0.009*
CD4+TLR-9+ [%] & sTLR-3 in serum [ng/mL]	0.514	2.873	0.009*
CD4+TLR-8+ [%] & CD19+TLR-9+ [%]	0.515	2.879	0.008*
CD19+TLR-7+ [%] & CD8+TLR-9+ [%]	0.515	2.879	0.008*
CD19+TLR-8+ [%] & sTLR-3 in serum [ng/mL]	0.516	2.890	0.008*
CD4+TLR-2+ [%] & CD19+TLR-7+ [%]	0.517	2.897	0.008*
CD19+TLR-3+ [%] & CD8+TLR-4+ [%]	0.518	2.908	0.008*
CD4+TLR-2+ [%] & CD4+TLR-8+ [%]	0.519	2.914	0.008*
CD8+TLR-4+ [%] & CD4+TLR-7+ [%]	0.520	2.919	0.008*
CD19+TLR-2+ [%] & sTLR-8 in serum [ng/mL]	0.524	2.952	0.007*
CD4+TLR-4+ [%] & sTLR-9 in serum [ng/mL]	0.526	2.968	0.007*
CD19+TLR-2+ [%] & CD19+TLR-8+ [%]	0.528	2.982	0.007*
CD4+TLR-7+ [%] & sTLR-8 in serum [ng/mL]	0.532	3.012	0.006*
sTLR-2 in serum [ng/mL] & sTLR-8 in serum [ng/mL]	0.532	3.012	0.006*
CD19+TLR-7+ [%] & CD19+TLR-9+ [%]	0.532	3.014	0.006*
CD8+TLR-3+ [%] & CD19+TLR-9+ [%]	0.533	3.021	0.006*
CD4+TLR-8+ [%] & CD8+TLR-9+ [%]	0.541	3.084	0.005*
CD8+TLR-7+ [%] & sTLR-9 in serum [ng/mL]	0.544	3.105	0.005*
CD4+TLR-3+ [%] & sTLR-8 in serum [ng/mL]	0.545	3.115	0.005*
CD4+TLR-7+ [%] & CD19+TLR-9+ [%]	0.545	3.121	0.005*

CD8+TLR-9+ [%] & sTLR-8 in serum [ng/mL]	0.547	3.130	0.005*
CD8+TLR-3+ [%] & CD8+TLR-8+ [%]	0.550	3.154	0.004*
CD8+TLR-4+ [%] & CD8+TLR-7+ [%]	0.554	3.195	0.004*
CD8+TLR-4+ [%] & CD8+TLR-8+ [%]	0.556	3.210	0.004*
CD19+TLR-7+ [%] & sTLR-8 in serum [ng/mL]	0.561	3.249	0.004*
CD19+TLR-4+ [%] & CD4+TLR-9+ [%]	0.563	3.269	0.003*
CD4+TLR-8+ [%] & sTLR-8 in serum [ng/mL]	0.563	3.271	0.003*
CD4+TLR-3+ [%] & CD4+TLR-4+ [%]	0.566	3.288	0.003*
sTLR-4 in serum [ng/mL] & sTLR-8 in serum [ng/mL]	0.567	3.304	0.003*
CD8+TLR-2+ [%] & sTLR-8 in serum [ng/mL]	0.568	3.308	0.003*
CD19+TLR-4+ [%] & sTLR-2 in serum [ng/mL]	0.569	3.321	0.003*
CD8+TLR-3+ [%] & sTLR-4 in serum [ng/mL]	0.571	3.333	0.003*
CD8+TLR-8+ [%] & sTLR-9 in serum [ng/mL]	0.574	3.364	0.003*
CD4+TLR-8+ [%] & sTLR-4 in serum [ng/mL]	0.579	3.402	0.002*
CD19+TLR-3+ [%] & CD19+TLR-9+ [%]	0.581	3.423	0.002*
CD4+TLR-3+ [%] & CD4+TLR-9+ [%]	0.581	3.424	0.002*
CD19+TLR-2+ [%] & CD4+TLR-9+ [%]	0.582	3.429	0.002*
CD19+TLR-8+ [%] & sTLR-2 in serum [ng/mL]	0.586	3.470	0.002*
CD4+TLR-2+ [%] & CD4+TLR-7+ [%]	0.587	3.481	0.002*
CD8+TLR-3+ [%] & CD19+TLR-3+ [%]	0.588	3.487	0.002*
CD4+TLR-2+ [%] & sTLR-2 in serum [ng/mL]	0.589	3.499	0.002*
CD8+TLR-9+ [%] & CD19+TLR-3+ [%]	0.589	3.499	0.002*
CD19+TLR-2+ [%] & CD19+TLR-9+ [%]	0.591	3.513	0.002*
CD4+TLR-2+ [%] & CD19+TLR-3+ [%]	0.592	3.520	0.002*
CD8+TLR-4+ [%] & CD19+TLR-4+ [%]	0.596	3.562	0.002*
CD19+TLR-2+ [%] & CD4+TLR-8+ [%]	0.602	3.613	0.001*
CD4+TLR-3+ [%] & CD19+TLR-9+ [%]	0.603	3.625	0.001*
CD8+TLR-2+ [%] & CD19+TLR-9+ [%]	0.607	3.661	0.001*
CD4+TLR-9+ [%] & sTLR-4 in serum [ng/mL]	0.610	3.694	0.001*
CD4+TLR-2+ [%] & CD8+TLR-9+ [%]	0.613	3.723	0.001*
sTLR-3 in serum [ng/mL] & sTLR-9 in serum [ng/mL]	0.614	3.726	0.001*
CD4+TLR-2+ [%] & CD4+TLR-3+ [%]	0.615	3.743	0.001*
CD8+TLR-3+ [%] & CD4+TLR-8+ [%]	0.616	3.752	0.001*
sTLR-2 in serum [ng/mL] & sTLR-7 in serum [ng/mL]	0.617	3.760	0.001*
CD4+TLR-7+ [%] & sTLR-4 in serum [ng/mL]	0.619	3.776	0.001*
CD19+TLR-3+ [%] & sTLR-8 in serum [ng/mL]	0.620	3.787	0.001*
CD8+TLR-4+ [%] & sTLR-3 in serum [ng/mL]	0.621	3.799	0.001*
CD4+TLR-3+ [%] & sTLR-9 in serum [ng/mL]	0.621	3.803	0.001*
CD19+TLR-2+ [%] & CD4+TLR-4+ [%]	0.624	3.831	0.001*
CD8+TLR-2+ [%] & sTLR-9 in serum [ng/mL]	0.629	3.877	0.001*
CD4+TLR-8+ [%] & sTLR-2 in serum [ng/mL]	0.629	3.885	0.001*
CD8+TLR-3+ [%] & CD4+TLR-7+ [%]	0.630	3.893	0.001*
CD19+TLR-7+ [%] & sTLR-2 in serum [ng/mL]	0.631	3.906	0.001*
CD19+TLR-2+ [%] & CD19+TLR-7+ [%]	0.632	3.910	0.001*
CD8+TLR-7+ [%] & CD8+TLR-9+ [%]	0.634	3.934	0.001*
sTLR-7 in serum [ng/mL] & sTLR-8 in serum [ng/mL]	0.636	3.954	0.001*
CD4+TLR-4+ [%] & CD19+TLR-4+ [%]	0.638	3.978	0.001*
CD19+TLR-9+ [%] & sTLR-8 in serum [ng/mL]	0.641	4.008	0.001*
CD19+TLR-2+ [%] & sTLR-9 in serum [ng/mL]	0.642	4.016	0.001*
CD8+TLR-2+ [%] & CD4+TLR-9+ [%]	0.642	4.019	0.001*
CD8+TLR-8+ [%] & CD19+TLR-8+ [%]	0.645	4.050	0.000*
CD4+TLR-3+ [%] & CD8+TLR-4+ [%]	0.645	4.052	0.000*
CD19+TLR-9+ [%] & sTLR-7 in serum [ng/mL]	0.648	4.078	0.000*
CD4+TLR-4+ [%] & sTLR-2 in serum [ng/mL]	0.648	4.081	0.000*
sTLR-8 in serum [ng/mL] & sTLR-9 in serum [ng/mL]	0.650	4.098	0.000*
CD4+TLR-4+ [%] & CD8+TLR-9+ [%]	0.651	4.110	0.000*
CD4+TLR-2+ [%] & sTLR-8 in serum [ng/mL]	0.651	4.116	0.000*
CD4+TLR-8+ [%] & sTLR-3 in serum [ng/mL]	0.656	4.173	0.000*
CD4+TLR-2+ [%] & CD19+TLR-9+ [%]	0.657	4.181	0.000*
CD4+TLR-7+ [%] & sTLR-7 in serum [ng/mL]	0.659	4.206	0.000*
CD19+TLR-2+ [%] & sTLR-7 in serum [ng/mL]	0.663	4.245	0.000*
CD8+TLR-2+ [%] & CD19+TLR-4+ [%]	0.663	4.248	0.000*

CD8+TLR-3+ [%] & CD19+TLR-7+ [%]	0.664	4.260	0.000*
CD8+TLR-2+ [%] & sTLR-7 in serum [ng/mL]	0.666	4.282	0.000*
CD8+TLR-4+ [%] & sTLR-4 in serum [ng/mL]	0.666	4.287	0.000*
CD19+TLR-9+ [%] & CD8+TLR-8+ [%]	0.666	4.287	0.000*
sTLR-4 in serum [ng/mL] & sTLR-9 in serum [ng/mL]	0.667	4.290	0.000*
CD8+TLR-2+ [%] & CD8+TLR-8+ [%]	0.667	4.292	0.000*
CD8+TLR-2+ [%] & CD4+TLR-3+ [%]	0.669	4.321	0.000*
CD4+TLR-3+ [%] & sTLR-4 in serum [ng/mL]	0.670	4.328	0.000*
CD8+TLR-9+ [%] & sTLR-4 in serum [ng/mL]	0.672	4.350	0.000*
CD4+TLR-7+ [%] & CD8+TLR-9+ [%]	0.673	4.363	0.000*
CD8+TLR-2+ [%] & sTLR-4 in serum [ng/mL]	0.674	4.378	0.000*
CD4+TLR-8+ [%] & sTLR-7 in serum [ng/mL]	0.677	4.408	0.000*
CD19+TLR-7+ [%] & sTLR-3 in serum [ng/mL]	0.679	4.439	0.000*
CD4+TLR-2+ [%] & CD19+TLR-2+ [%]	0.681	4.461	0.000*
CD8+TLR-8+ [%] & sTLR-8 in serum [ng/mL]	0.682	4.473	0.000*
CD8+TLR-8+ [%] & CD8+TLR-9+ [%]	0.684	4.498	0.000*
CD8+TLR-7+ [%] & CD19+TLR-9+ [%]	0.693	4.612	0.000*
CD19+TLR-2+ [%] & CD8+TLR-4+ [%]	0.695	4.635	0.000*
CD8+TLR-2+ [%] & CD4+TLR-4+ [%]	0.695	4.638	0.000*
sTLR-2 in serum [ng/mL] & CD19+TLR-3+ [%]	0.698	4.675	0.000*
CD8+TLR-7+ [%] & CD4+TLR-8+ [%]	0.705	4.773	0.000*
CD4+TLR-7+ [%] & sTLR-2 in serum [ng/mL]	0.709	4.824	0.000*
CD8+TLR-7+ [%] & sTLR-2 in serum [ng/mL]	0.716	4.922	0.000*
CD4+TLR-7+ [%] & CD8+TLR-7+ [%]	0.718	4.950	0.000*
CD19+TLR-7+ [%] & CD19+TLR-8+ [%]	0.722	5.008	0.000*
CD19+TLR-9+ [%] & sTLR-3 in serum [ng/mL]	0.723	5.025	0.000*
CD8+TLR-7+ [%] & CD19+TLR-7+ [%]	0.729	5.103	0.000*
CD4+TLR-3+ [%] & CD19+TLR-8+ [%]	0.731	5.133	0.000*
sTLR-4 in serum [ng/mL] & sTLR-7 in serum [ng/mL]	0.738	5.238	0.000*
CD8+TLR-2+ [%] & CD8+TLR-9+ [%]	0.746	5.377	0.000*
CD4+TLR-4+ [%] & CD4+TLR-9+ [%]	0.751	5.453	0.000*
CD8+TLR-2+ [%] & sTLR-2 in serum [ng/mL]	0.759	5.597	0.000*
CD19+TLR-3+ [%] & sTLR-7 in serum [ng/mL]	0.761	5.620	0.000*
CD4+TLR-4+ [%] & CD8+TLR-4+ [%]	0.764	5.679	0.000*
CD8+TLR-2+ [%] & CD8+TLR-7+ [%]	0.766	5.716	0.000*
CD19+TLR-9+ [%] & sTLR-4 in serum [ng/mL]	0.774	5.867	0.000*
CD19+TLR-2+ [%] & CD8+TLR-7+ [%]	0.775	5.885	0.000*
CD4+TLR-3+ [%] & CD8+TLR-9+ [%]	0.778	5.937	0.000*
sTLR-3 in serum [ng/mL] & sTLR-4 in serum [ng/mL]	0.779	5.956	0.000*
CD19+TLR-2+ [%] & CD8+TLR-8+ [%]	0.790	6.189	0.000*
CD4+TLR-3+ [%] & CD8+TLR-7+ [%]	0.795	6.285	0.000*
CD19+TLR-9+ [%] & sTLR-2 in serum [ng/mL]	0.795	6.287	0.000*
CD8+TLR-9+ [%] & sTLR-9 in serum [ng/mL]	0.798	6.342	0.000*
CD19+TLR-2+ [%] & sTLR-2 in serum [ng/mL]	0.800	6.405	0.000*
CD4+TLR-3+ [%] & sTLR-2 in serum [ng/mL]	0.805	6.504	0.000*
CD4+TLR-2+ [%] & CD8+TLR-8+ [%]	0.806	6.527	0.000*
CD4+TLR-8+ [%] & CD8+TLR-8+ [%]	0.812	6.673	0.000*
CD4+TLR-9+ [%] & sTLR-9 in serum [ng/mL]	0.813	6.697	0.000*
CD8+TLR-8+ [%] & sTLR-7 in serum [ng/mL]	0.817	6.784	0.000*
CD19+TLR-3+ [%] & CD8+TLR-7+ [%]	0.817	6.799	0.000*
CD19+TLR-7+ [%] & CD8+TLR-8+ [%]	0.821	6.897	0.000*
CD8+TLR-9+ [%] & sTLR-2 in serum [ng/mL]	0.827	7.066	0.000*
CD19+TLR-9+ [%] & sTLR-9 in serum [ng/mL]	0.828	7.093	0.000*
CD19+TLR-2+ [%] & sTLR-4 in serum [ng/mL]	0.830	7.129	0.000*
CD8+TLR-2+ [%] & CD19+TLR-2+ [%]	0.833	7.209	0.000*
CD4+TLR-3+ [%] & sTLR-3 in serum [ng/mL]	0.833	7.209	0.000*
CD4+TLR-3+ [%] & CD19+TLR-7+ [%]	0.833	7.214	0.000*
CD8+TLR-2+ [%] & sTLR-3 in serum [ng/mL]	0.833	7.220	0.000*
CD8+TLR-4+ [%] & sTLR-9 in serum [ng/mL]	0.834	7.244	0.000*
sTLR-2 in serum [ng/mL] & sTLR-9 in serum [ng/mL]	0.835	7.288	0.000*
CD19+TLR-2+ [%] & CD4+TLR-3+ [%]	0.847	7.649	0.000*
CD19+TLR-2+ [%] & sTLR-3 in serum [ng/mL]	0.847	7.656	0.000*

CD4+TLR-2+ [%] & sTLR-7 in serum [ng/mL]	0.855	7.910	0.000*
CD4+TLR-2+ [%] & CD8+TLR-7+ [%]	0.856	7.944	0.000*
CD4+TLR-7+ [%] & CD8+TLR-8+ [%]	0.857	7.969	0.000*
CD4+TLR-3+ [%] & CD8+TLR-8+ [%]	0.860	8.067	0.000*
CD4+TLR-3+ [%] & CD4+TLR-8+ [%]	0.861	8.123	0.000*
CD19+TLR-3+ [%] & CD8+TLR-8+ [%]	0.864	8.216	0.000*
CD4+TLR-9+ [%] & sTLR-2 in serum [ng/mL]	0.864	8.216	0.000*
CD4+TLR-3+ [%] & CD19+TLR-3+ [%]	0.884	9.079	0.000*
CD8+TLR-7+ [%] & CD8+TLR-8+ [%]	0.892	9.460	0.000*
CD4+TLR-2+ [%] & sTLR-3 in serum [ng/mL]	0.894	9.552	0.000*
sTLR-3 in serum [ng/mL] & sTLR-7 in serum [ng/mL]	0.895	9.612	0.000*
CD8+TLR-8+ [%] & sTLR-3 in serum [ng/mL]	0.900	9.893	0.000*

CD8+TLR-4+ [%] & sTLR-2 in serum [ng/mL]	0.908	10.364	0.000*
CD19+TLR-3+ [%] & CD4+TLR-7+ [%]	0.916	10.934	0.000*
CD4+TLR-7+ [%] & CD19+TLR-7+ [%]	0.917	11.034	0.000*
CD4+TLR-3+ [%] & CD4+TLR-7+ [%]	0.943	13.633	0.000*
CD8+TLR-7+ [%] & sTLR-7 in serum [ng/mL]	0.945	13.867	0.000*
CD8+TLR-4+ [%] & CD4+TLR-9+ [%]	0.951	14.701	0.000*
CD19+TLR-3+ [%] & CD4+TLR-8+ [%]	0.954	15.221	0.000*
CD4+TLR-7+ [%] & CD4+TLR-8+ [%]	0.958	15.980	0.000*
CD8+TLR-7+ [%] & sTLR-3 in serum [ng/mL]	0.971	19.313	0.000*
CD19+TLR-7+ [%] & CD4+TLR-8+ [%]	0.974	20.459	0.000*
CD19+TLR-3+ [%] & CD19+TLR-7+ [%]	0.981	23.955	0.000*
